# A review of Canadian and Alaskan species of the genus *Liogluta* Thomson, and descriptions of three new species (Coleoptera, Staphylinidae, Aleocharinae)

**DOI:** 10.3897/zookeys.573.7878

**Published:** 2016-03-24

**Authors:** Jan Klimaszewski, Reginald P. Webster, David W. Langor, Derek Sikes, Caroline Bourdon, Benoit Godin, Crystal Ernst

**Affiliations:** 1 Natural Resources Canada, Canadian Forest Service, Laurentian Forestry Centre, 1055 du P.E.P.S., P.O. Box 10380, Stn. Sainte-Foy, Québec, Québec, Canada G1V 4C7; 2 24 Mill Stream Drive, Charters Settlement, New Brunswick, Canada E3C 1X1; 3 Natural Resources Canada, Canadian Forest Service, Northern Forestry Centre, 5320-122 Street NW, Edmonton, Alberta, Canada T6H 3S5; 4 University of Alaska Museum, 907 Yukon Dr., Fairbanks, Alaska, USA, 99775-6960; 5 14 A Thomson Rd., Whitehorse, Yukon, Canada Y1A 0C4; 6 Earth to Oceans Research Group, Department of Biological Sciences, Simon Fraser University, Burnaby, British Columbia, Canada, V5A 1S6

**Keywords:** Aleocharinae, Coleoptera, *Liogluta*, taxonomic review, Canada, Alaska

## Abstract

Fourteen species of *Liogluta* Thomson are reported from Canada and Alaska. Three of these are described as new to science: *Liogluta castoris* Klimaszewski & Webster, **sp. n.**; *Liogluta microgranulosa* Klimaszewski & Webster, **sp. n.**; and *Liogluta pseudocastoris* Klimaszewski & Webster, **sp. n.** The previously unknown male of *Liogluta gigantea* Klimaszewski & Langor, *Liogluta quadricollis* (Casey), *Liogluta wickhami* (Casey), and female of *Liogluta granulosa* Lohse are described, and illustrated. *Liogluta aloconotoides* Lohse is synonymized with *Liogluta terminalis* (Casey). New provincial and state records are provided for six *Liogluta* species. A key to species, revised distribution with new provincial records, and new natural history data are provided.

## Introduction

Casey (1894, 1906) described four species of *Liogluta* Thomson from British Columbia, Canada. Three of these were originally described in the genus *Anepsiota* Casey, and one in the genus *Athetota* Casey. Lohse et al. (1990) treated arctic Aleocharinae in North America and recorded four *Liogluta* species, three of which were described as new to science. Klimaszewski & Langor in Klimaszewski et al. (2011) reported three species of *Liogluta* from Newfoundl and Labrador, two of which were described as new to science. Gusarov (2003a), in his on-line catalogue of athetine species of America north of Mexico, listed 17 valid species of *Liogluta*, 12 of which occurred in Canada and Alaska. Bousquet et al. (2013) listed 12 species of *Liogluta* in Canada and Alaska. Since then, we have discovered three new species of *Liogluta* from Canada. There are presently 14 species in this genus occurring in Canada and Alaska.

## Materials and methods

All specimens in this study were dissected to examine the genital structures to aid with identification. Extracted genital structures were dehydrated in absolute alcohol, mounted in Canada balsam on celluloid micro-slides, and pinned with the specimens from which they originated. Images of the entire body and the genital structures were taken using an image processing system (Nikon SMZ 1500 stereoscopic microscope; Nikon Digital Camera DXM 1200F, and Adobe Photoshop software).

Morphological terminology mainly follows that used by Seevers (1978) and Klimaszewski et al. (2011). The ventral side of the median lobe of the aedeagus is considered to be the side of the bulbus containing the foramen mediale, the entrance of the ductus ejaculatorius, and the adjacent ventral side of the tubus of the median lobe with the internal sac and its structures (this part is referred to as the parameral side in some recent publications); the opposite side is referred to as the dorsal aspect. In species descriptions, microsculpture refers to the surface of the upper forebody (head, pronotum, and elytra).

### Depository/institutional abbreviations

CNC
Canadian National Collection of Insects, Arachnids, and Nematodes, Agriculture and Agri-Food Canada, Ottawa, Ontario, Canada 


FMNH
 The Field Museum, Chicago, Illinois, USA 


LFC
 Natural Resources Canada, Canadian Forest Service, Laurentian Forestry Centre, R. Martineau Insectarium, Québec City, Quebec, Canada 


NBM
 New Brunswick Museum, Saint John, New Brunswick, Canada 


NoFC
 Natural Resources Canada, Canadian Forest Service, Northern Forestry Centre, Arthropod Museum, Edmonton, Alberta, Canada 


RWC
 Reginald Webster Collection, Charters Settlement, New Brunswick, Canada 


UAM
University of Alaska Museum, University of Alaska, Fairbanks, Alaska, USA 


USNM
 United States National Museum, Smithsonian Institution, Washington, D.C., USA 


ZMH
Museum of Zoology, Helsinki, Finland 


### Abbreviations of Canadian provinces and territories

AB
 – Alberta 


BC
 – British Columbia 


LB
 – Labrador 


MB
 – Manitoba 


NB
 – New Brunswick 


NF
 – Newfoundland 


NS
 – Nova Scotia 


NT
 – Northwest Territories 


NU
 – Nunavut 


ON
 – Ontario 


PE
 – Prince Edward Island 


QC
 – Quebec 


SK
 – Saskatchewan 


YT
 – Yukon Territory 


USA state abbreviations follow those of the US Postal Service.

### Checklist of Canadian and Alaskan *Liogluta* species

New species and new jurisdictional records are indicated in **bold** type.

**Genus *Liogluta* Thomson, 1858**

***Terminalis* species group**

1. *Liogluta terminalis* (Casey, 1906).

*Liogluta aloconotoides* Lohse, 1990. **New synonymy**.

*Liogluta renominata* (Bernhauer & Scheerpeltz, 1926). Synonymized by Seevers (1978) Canada: LB, NB, NF, NS, ON, QC, AB, YT, BC. USA: **MT**, **NH**.

2. *Liogluta quadricollis* (Casey, 1894). Canada: BC.

3. *Liogluta trapezicollis* Lohse, 1990. Canada: **BC**, YT. USA: **AK**.

4. *Liogluta wickhami* (Casey, 1894). Canada: BC.

5. *Liogluta vasta* (Mӓklin, 1853). Canada: YT?. USA: AK.

***Nigropolita* species group**

6. *Liogluta nigropolita* (Bernhauer, 1907). Canada: LB, NF, **NT**, NU, QC, YT. USA: NH.

7. *Liogluta nitens* (Mӓklin, 1852). Canada: **AB**, BC, YT. USA: AK, OR, WA.

*Liogluta apposita* (Casey, 1911). Synonymized by Gusarov (2003a).

*Liogluta insolens* (Casey, 1910). Synonymized by Gusarov (2003a).

*Liogluta resplendens* (Casey, 1910). Synonymized by Gusarov (2003a).

***Granulosa* species group**

8. *Liogluta granulosa* Lohse, 1990. Canada: YT. USA: AK.

***Microgranulosa* species group**

9. *Liogluta atriventris* (Casey, 1906). Canada: BC.

10. ***Liogluta castoris*** Klimaszewski & Webster, **sp. n. Canada: NB, NS, QC.**

11. *Liogluta intermedia* Klimaszewski & Langor, 2011. Canada: **LB**, NF, **NS**, **QC**, **ON.** USA: **NH.**

12. ***Liogluta microgranulosa*** Klimaszewski & Webster, **sp. n. Canada: NB.**

13. ***Liogluta pseudocastoris*** Klimaszewski & Webster, **sp. n. Canada: NB.**

***Gigantea* species group**

14. *Liogluta gigantea* Klimaszewski & Langor, 2011. Canada: LB, **QC, ON.**

## Species excluded from *Liogluta*

*Homalota aemula* Erichson, 1839: 102. Considered as Atheta (Liogluta) by Bernhauer (1907: 394) up to Moore and Legner (1975: 354), as *Liogluta* by Seevers (1978: 263). Treated as *Atheta* by Gusarov (2003b).

Atheta (Lamiota) keeni Casey, 1910: 17. Considered as Atheta (Liogluta) by Fenyes (1920: 209) up to Moore and Legner (1975: 364). Treated as *Lamiota* by Seevers (1978: 112, 263), as *Atheta* by Gusarov (2003b).

Atheta (Lamiota) achromata Casey, 1911: 82. Considered as Atheta (Liogluta) up to Moore and Legner (1975: 353). Treated as *Lamiota* by Seevers (1978: 263), as *Atheta* by Gusarov (2003b), and synonymized with *Atheta keeni* Casey.

Atheta (Lamiota) profecta Casey, 1911: 83. Considered as Atheta (Liogluta) by Fenyes (1920: 209) up to Moore and Legner (1975: 370). Treated as *Lamiota* by Seevers (1978: 263), as *Atheta* by Gusarov (2003b), and synonymized with *Atheta keeni* Casey.

### Taxonomic review

#### Liogluta

Taxon classificationAnimaliaColeopteraStaphylinidae

Thomson, 1858

Liogluta Thomson, 1858: 35. Type species Homalota umbonata Erichson, 1839, by monotypy. As valid genus: Lohse (1974); Lohse et al. (1990).
Anepsiota Casey, 1894: 321; Casey (1906: 335); as Atheta (Anepsiota): Casey (1910: 12), Fenyes (1920: 203). Synonymized by Bernhauer and Scheerpeltz (1926: 656); Moore and Legner (1975: 350).
Athetota Casey, 1906: 334. Synonymized with Atheta (Anepsiota) by Fenyes (1920: 203); as synonym of Atheta (Liogluta): Bernhauer and Scheerpeltz (1926: 656), Moore and Legner (1975: 350).
Hypnota Mulsant & Rey, 1873: 591. Synonymized with Atheta (Liogluta) by Fenyes (1920: 203); Bernhauer and Scheerpeltz (1926: 656); Moore and Legner (1975: 350).

##### Diagnosis.

Body length ranging from 2.8–5.4 mm, body narrowly subparallel (Figs 1, 8, 16, 23, 30, 35, 44, 50, 58, 65, 72, 79, 86, 90), moderately flattened; elytra and abdomen wider than head and pronotum; uniformly dark brown or reddish-brown with head and posterior abdomen dark brown to almost black; integument of forebody with strong meshed microsculpture; surface of elytra often granulose (Figs 50, 51); head with incomplete postocular carinae, postocular region of head long and in most species longer than diameter of eye; glossae split into two lobes; antennae long with articles VI-X subquadrate, slightly transverse or rarely slightly elongate; pronotum with hypomera fully visible in lateral view; pubescence of pronotum directed posteriad on midline of disc and posterolaterad to laterad on sides; mesocoxae narrowly separated, metasternal process short and acute; legs long, three basal articles of metatarsi highly elongate in most species; tarsal formula 5-5-5. **Male.** Apical margin of male tergite VIII often with broad, variably-shaped rectangular projection, with edge entire or bearing crenulation or variably shaped structures, with two lateral teeth and sometimes with additional median tooth; integument of disc often with broadly distributed asperate punctation near apex (Figs 3, 11, 18, 25, 38, 46, 53, 60, 67, 74, 81, 88, 92); median lobe of aedeagus simple with apical part variably shaped in lateral view (Figs 2, 9, 17, 24, 36, 45, 52, 59, 66, 73, 80, 87, 91). **Female.** Spermatheca with capsule narrowly club-shaped or tubular, with apical invagination moderate to deep, stem long, sinuate, looped or twisted posteriorly (Figs 7, 15, 22, 29, 34, 42, 43, 57, 64, 71, 78, 85, 96); sternite VIII with apical margin rounded or medially emarginate, antecostal suture arcuate, or slightly to distinctly sinuate (Figs 6, 14, 21, 28, 33, 41, 49, 56, 63, 70, 77, 84, 89, 95).

**Figures 1–7.**
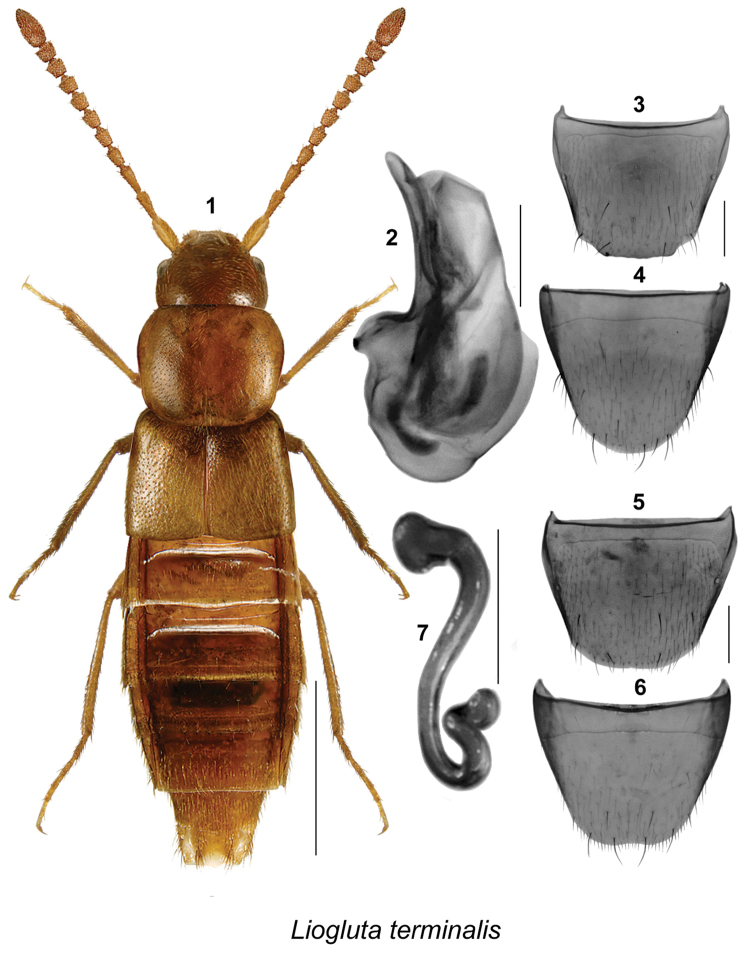
*Liogluta terminalis* (Casey): **1** habitus in dorsal view **2** median lobe of aedeagus in lateral view **3** male tergite VIII **4** male sternite VIII **5** female tergite VIII **6** female sternite VIII **7** spermatheca. Scale bar of habitus = 1 mm, remaining scale bars = 0.2 mm.

**Figures 8–15.**
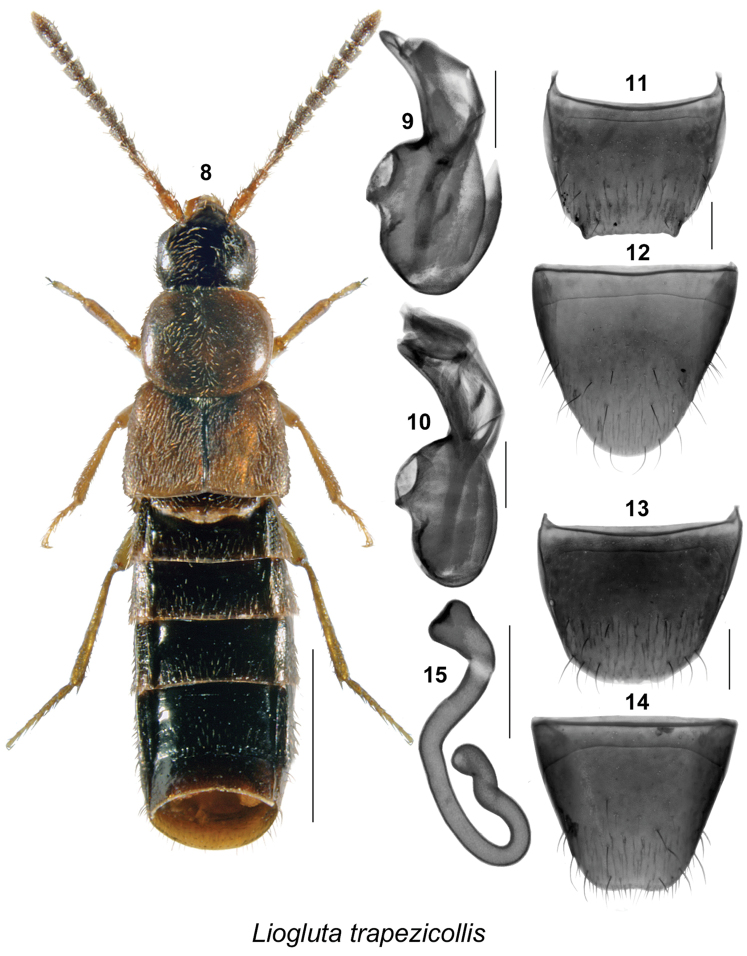
*Liogluta trapezicollis* Lohse: **8** habitus in dorsal view **9**, **10** median lobe of aedeagus in lateral view **11** male tergite VIII **12** male sternite VIII **13** female tergite VIII **14** female sternite VIII **15** spermatheca. Scale bar of habitus = 1 mm, remaining scale bars = 0.2 mm.

**Figures 16–22.**
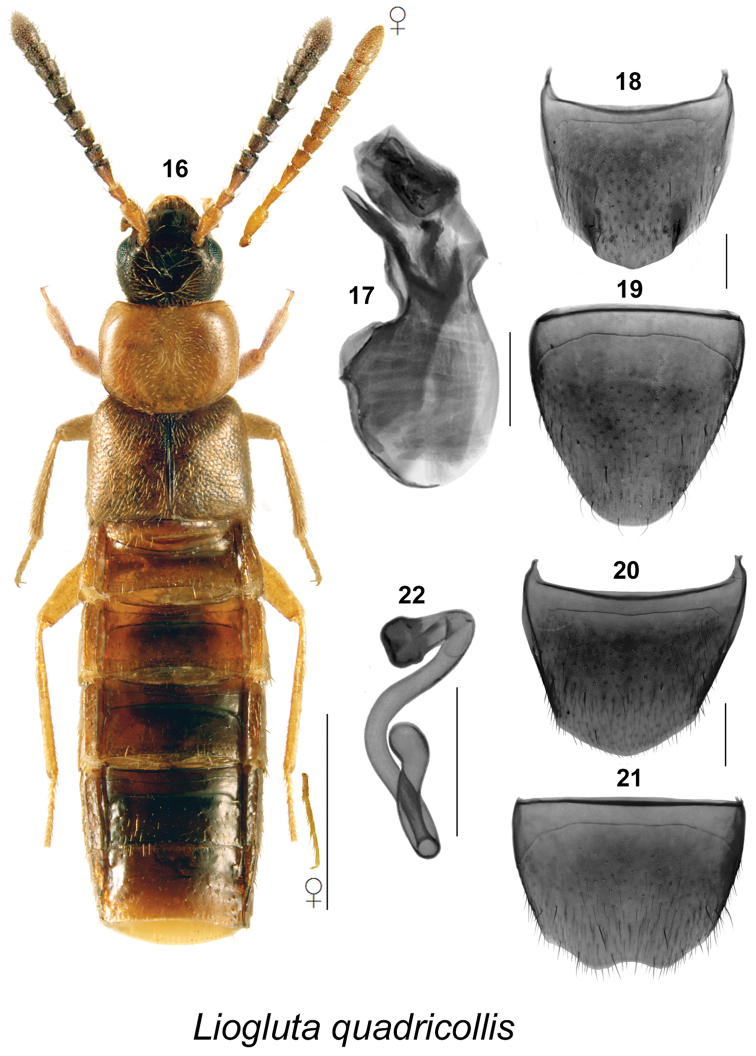
*Liogluta quadricollis* (Casey): **16** habitus in dorsal view **17** median lobe of aedeagus in lateral view **18** male tergite VIII **19** male sternite VIII [16–19 based on male from BC] **20** female tergite VIII **21** female sternite VIII **22** spermatheca (19–22 based on female holotype). Scale bar of habitus = 1 mm, remaining scale bars = 0.2 mm.

**Figures 23–29.**
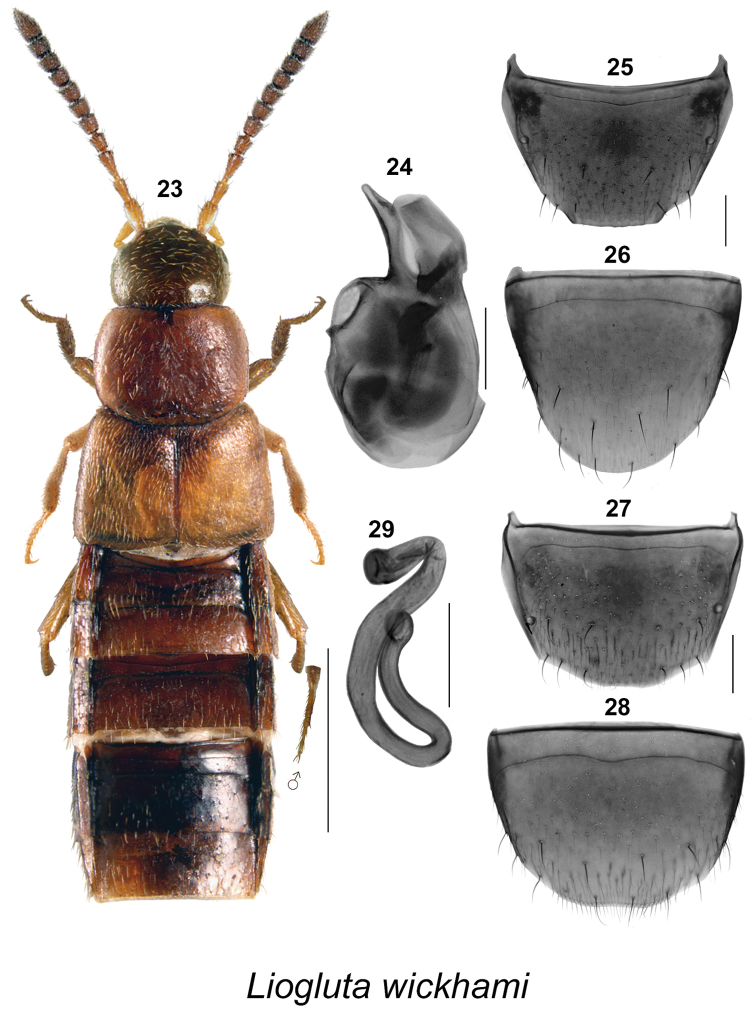
*Liogluta wickhami* (Casey): **23** habitus in dorsal view **24** median lobe of aedeagus in lateral view **25** male tergite VIII **26** male sternite VIII **27** female tergite VIII **28** female sternite VIII **29** spermatheca [**23**, **27–29** based on female holotype]. Scale bar of habitus = 1 mm, remaining scale bars = 0.2 mm.

**Figures 30–34.**
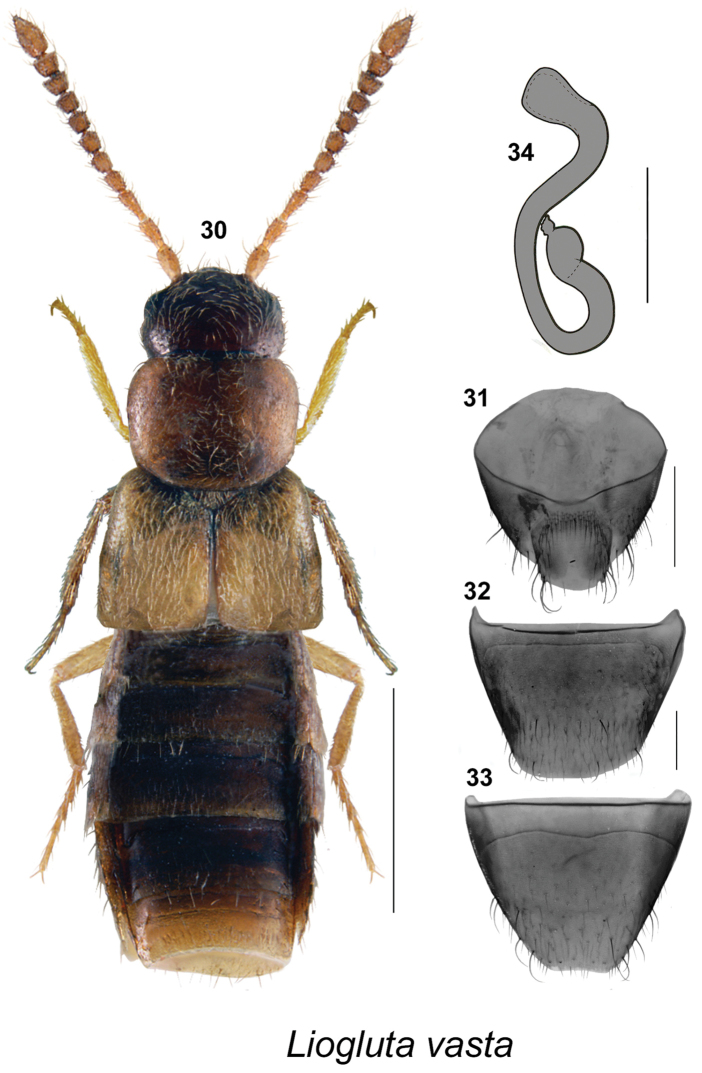
*Liogluta vasta* (Mäklin): **30** habitus in dorsal view **31** female pygidium (terminal segments) **32** female tergite VIII **33** female sternite VIII (30–33 based on female holotype) **34** spermatheca (based on YT specimen after Lohse and Smetana 1895). Scale bar of habitus = 1 mm, remaining scale bars = 0.2 mm.

**Figures 35–43.**
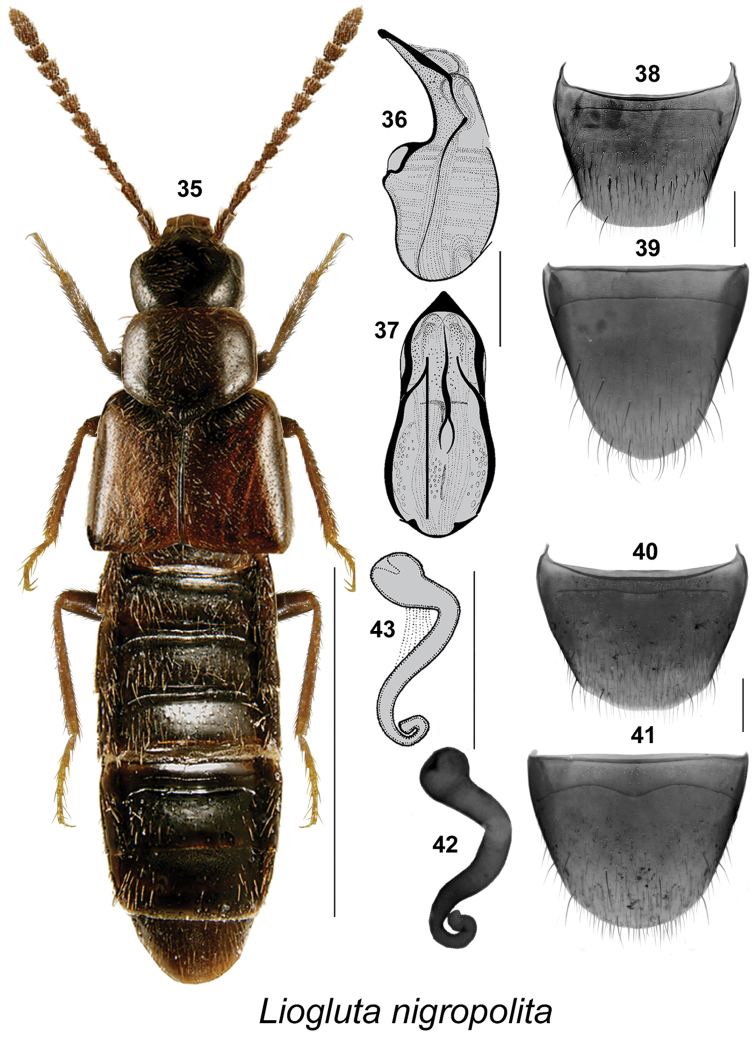
*Liogluta nigropolita* (Bernhauer): **35** habitus in dorsal view **36** median lobe of aedeagus in lateral view **37** median lobe of aedeagus in dorsal view (36, 37 after Lohse et al. 1990) **38** male tergite VIII **39** male sternite VIII **40** female tergite VIII **41** female sternite VIII **42, 43** spermatheca (43 after Lohse et al. 1990). Scale bar of habitus = 1 mm, remaining scale bars = 0.2 mm.

**Figures 44–49.**
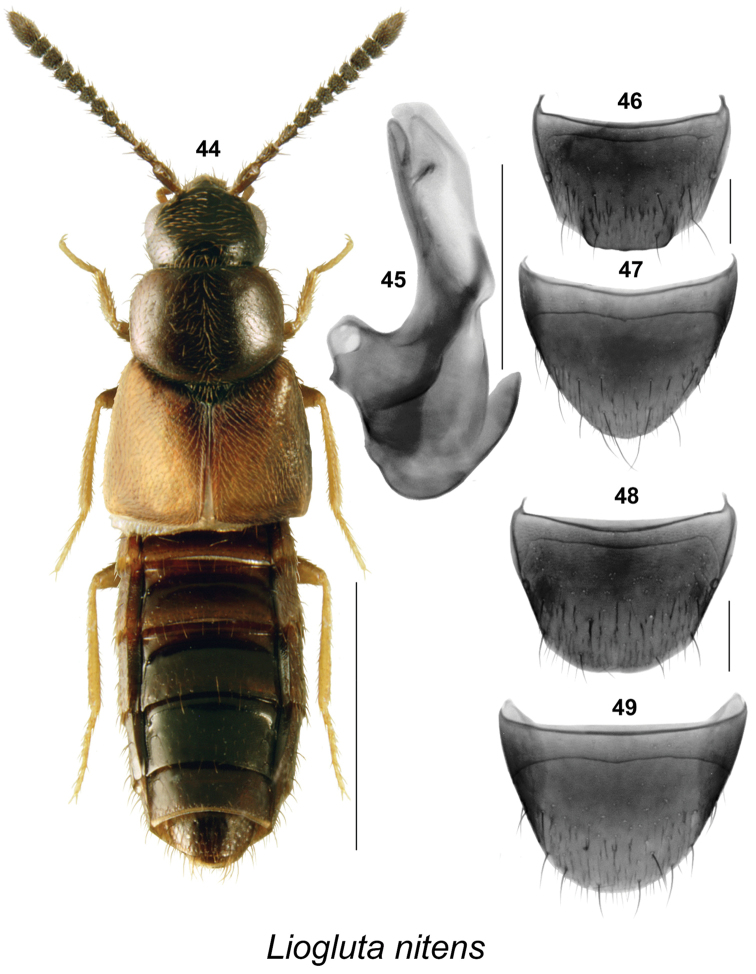
*Liogluta nitens* (Mäklin): **44** habitus in dorsal view (morphotype with broad and long elytra) **45** median lobe of aedeagus in lateral view **46** male tergite VIII **47** male sternite VIII **48** female tergite VIII **49** female sternite VIII. Scale bar of habitus = 1 mm, remaining scale bars = 0.2 mm.

**Figures 50–57.**
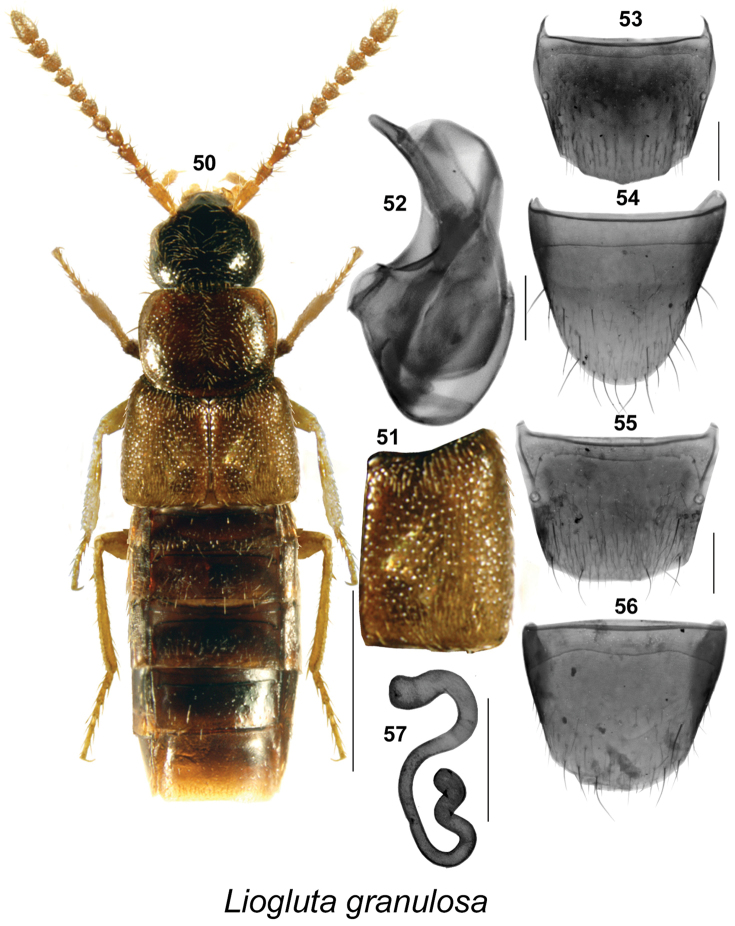
*Liogluta granulosa* Lohse: **50** habitus in dorsal view **51** elytron **52** median lobe of aedeagus in lateral view **53** male tergite VIII **54** male sternite VIII [50–54 based on male holotype] **55** female tergite VIII **56** female sternite VIII **57** spermatheca [**55–57** based on female from YT]. Scale bar of habitus = 1 mm, remaining scale bars = 0.2 mm.

**Figures 58–64.**
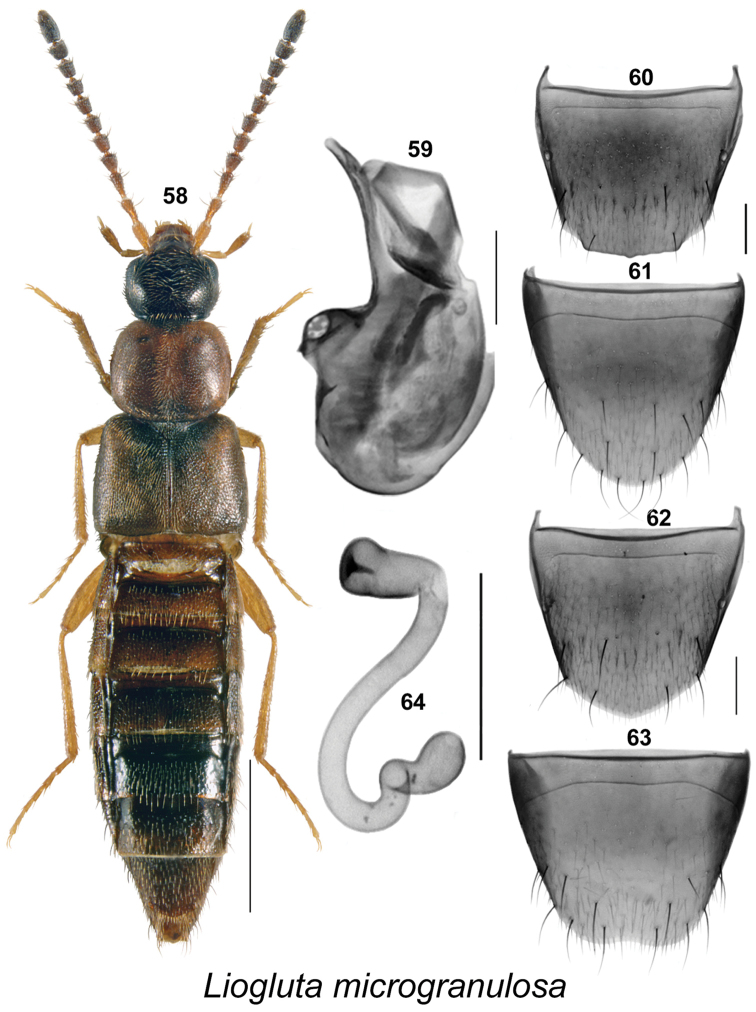
*Liogluta microgranulosa* Klimaszewski & Webster, sp. n.: **58** habitus in dorsal view **59** median lobe of aedeagus in lateral view **60** male tergite VIII **61** male sternite VIII **62** female tergite VIII **63** female sternite VIII **64** spermatheca (**58–64** based on type specimens). Scale bar of habitus = 1 mm, remaining scale bars = 0.2 mm.

**Figures 65–71.**
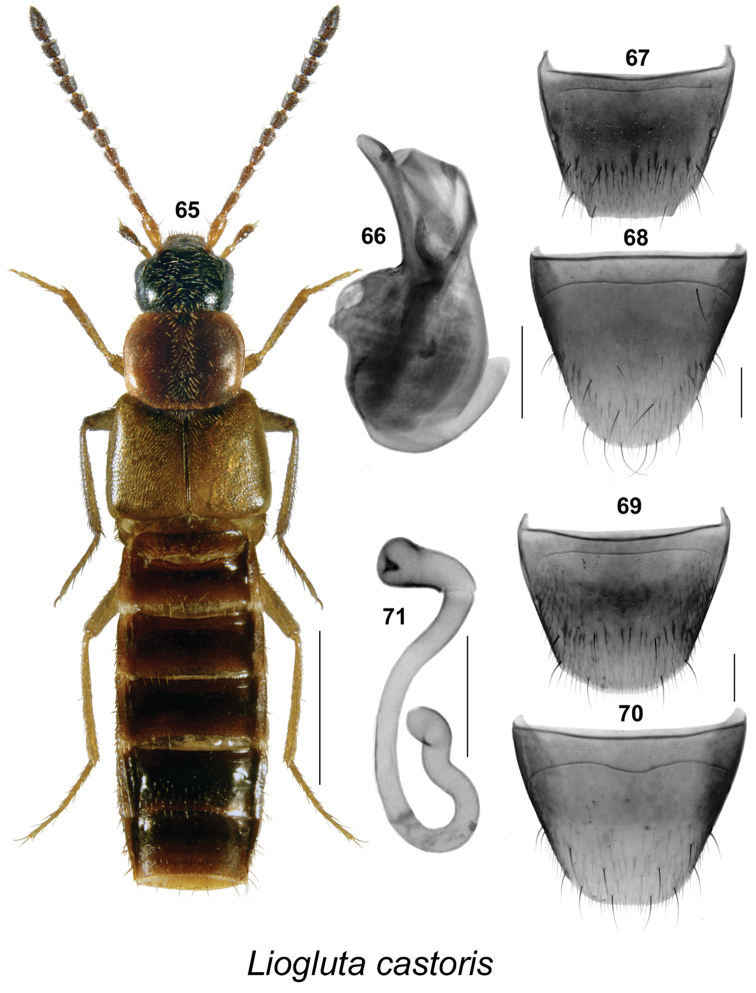
*Liogluta castoris* Klimaszewski & Webster, sp. n.: **65** habitus in dorsal view **66** median lobe of aedeagus in lateral view **67** male tergite VIII **68** male sternite VIII **69** female tergite VIII **70** female sternite VIII **71** spermatheca (**65–71** based on type specimens). Scale bar of habitus = 1 mm, remaining scale bars = 0.2 mm.

**Figures 72–78.**
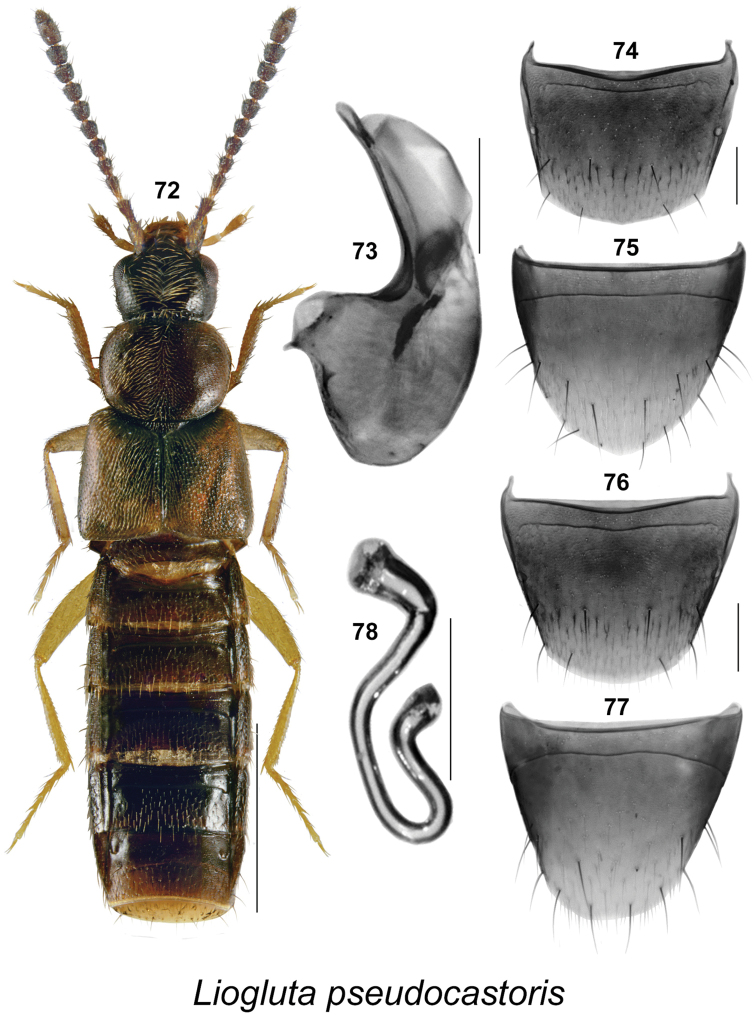
*Liogluta pseudocastoris* Klimaszewski & Webster, sp. n.: **72** habitus in dorsal view **73** median lobe of aedeagus in lateral view **74** male tergite VIII **75** male sternite VIII **76** female tergite VIII **77** female sternite VIII **78** spermatheca (**72–78** based on type specimens). Scale bar of habitus = 1 mm, remaining scale bars = 0.2 mm.

**Figures 79–85.**
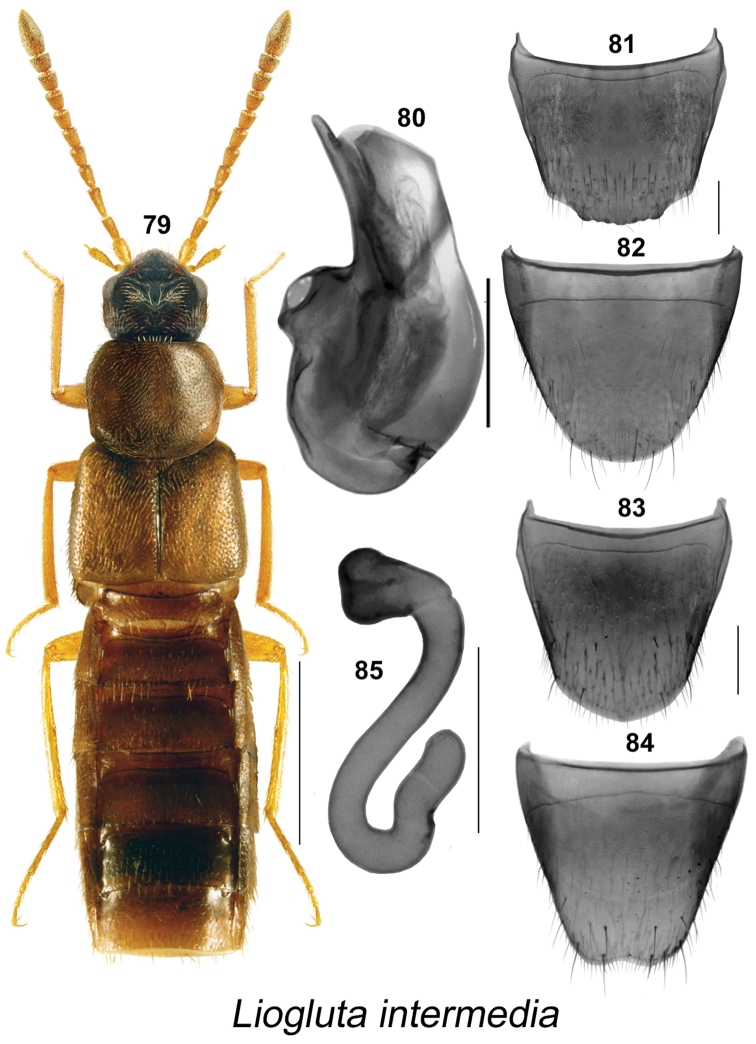
*Liogluta intermedia* Klimaszewski & Langor: **79** habitus in dorsal view **80** median lobe of aedeagus in lateral view **81** male tergite VIII, **82** male sternite VIII **83** female tergite VIII **84** female sternite VIII **85** spermatheca. Scale bar of habitus = 1 mm, remaining scale bars = 0.2 mm.

**Figures 86–89.**
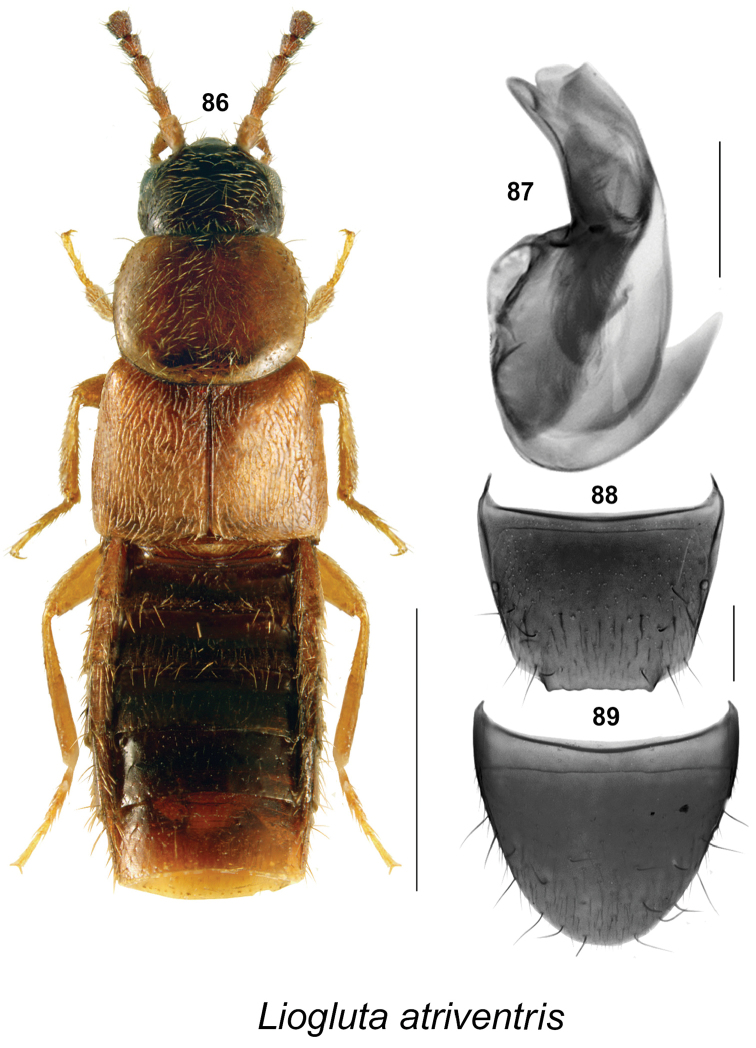
*Liogluta atriventris* (Casey): **86** habitus in dorsal view **87** median lobe of aedeagus in lateral view **88** male tergite VIII **89** male sternite VIII (**86–89** based on male lectotype). Female unknown. Scale bar of habitus = 1 mm, remaining scale bars = 0.2 mm.

**Figures 90–96.**
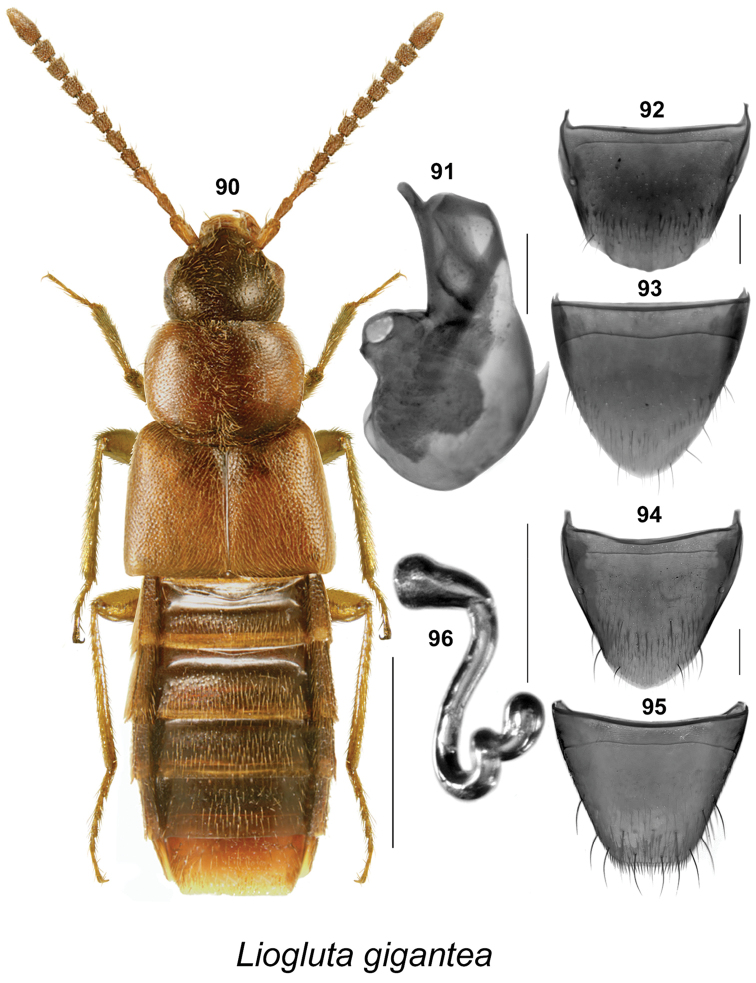
*Liogluta gigantea* Klimaszewski & Langor: **90** habitus in dorsal view **91** median lobe of aedeagus in lateral view **92** male tergite VIII **93** male sternite VIII **94** female tergite VIII **95** female sternite VIII **96** spermatheca (**90**, **94–96** based on holotype, **91–93** based on male from Ontario). Scale bar of habitus = 1 mm, remaining scale bars = 0.2 mm.

##### Distribution.

The genus is holarctic in distribution (Smetana 2004). Seevers (1978) mentioned a few species from Africa and Jamaica but these records need verification.

##### Key to Canadian and Alaskan species of *Liogluta*

**Table:** 

1	Eyes small, each shorter than postocular region of head in dorsal view (Figs 1, 8, 16, 23, 30)	**2**
–	Eyes large, each subequal in length to postocular region of head in dorsal view (Figs 35, 44, 50, 58, 65, 72, 79, 86, 90)	**6**
2	Antennae enlarged (Fig. 16)	***Liogluta quadricollis* (Casey)**
–	Antennae normally developed and not enlarged (Figs 1, 8, 23, 30)	**3**
3	Body more-or-less uniformly reddish- to yellowish-brown (Fig. 1)	***Liogluta terminalis* (Casey)**
–	Body brown to dark brown with paler pronotum and elytra, or reddish-yellow with brown head, antennae and abdomen (Figs 8, 23, 30)	**4**
4	Body narrow (Fig. 8); abdomen subparallel, distinctly narrower at base than elytra (Fig. 8); male tergite VIII truncate apically, with two large lateral teeth and apical margin denticulate (Fig. 11); bulbus moderately large, tubus long with apical part moderately broad (Figs 9, 10); spermatheca with broad capsule, with apical invagination small and shallow, stem sinuate with moderately long posterior loop (Fig. 15)	***Liogluta trapezicollis* Lohse**
–	Body broad (Figs 23, 30); abdomen arcuate laterally and broadest at middle of its length; genital structures shaped differently (Figs 24, 29, 34)	**5**
5	Pronotum about evenly arcuate laterally (Fig. 23); male tergite VIII truncate apically, with two small lateral teeth and apical margin slightly crenulate (Fig. 25); spermatheca with capsule narrow and apical invagination deep, stem sinuate with long posterior loop (Fig. 29)	***Liogluta wickhami* (Casey)**
–	Pronotum trapezoidal in shape, narrowest at base and broadest in apical third (Fig. 30); male unknown; spermatheca with broad capsule, with apical invagination small and shallow, stem sinuate with moderately long posterior loop (Fig. 34)	***Liogluta vasta* (Mäklin)**
6	Elytra broad, about 25% wider than maximum width of pronotum (Figs 35, 44, 90), integument moderately to highly glossy; male tergite VIII truncate apically and without distinct lateral teeth, not or moderately produced apically (Figs 38, 46, 92)	**7**
–	Elytra narrow, at most 20% wider than maximum width of pronotum (Figs 50, 58, 65, 72, 79, 86); integument moderately glossy in most species; male tergite VIII usually with well-defined lateral teeth, and with or without crenulation on apical margin (Figs 53, 60, 67, 74, 81, 88)	**9**
7	Body moderately glossy (Fig. 90); antennal articles VI-X subquadrate to slightly elongate (Fig. 90); terminalia and genitalia as illustrated (Figs 91–96)	***Liogluta gigantea* Klimaszewski & Langor**
–	Body highly glossy (Figs 35, 44); antennal articles VI-X slightly to distinctly transverse (Figs 35, 44); terminalia and genitalia shaped differently (Figs 36–43, 45–49)	**8**
8	Antennal articles VI-X subquadrate to slightly transverse (Fig. 35); body usually uniformly dark brown to black (Fig. 35); male tergite VIII truncate and not produced apically (Fig. 38); remaining terminalia, median lobe of aedeagus, and spermatheca as illustrated (Figs 36, 37, 39–43)	***Liogluta nigropolita* (Bernhauer)**
–	Antennal articles VI-X distinctly transverse (Fig. 44); body dark brown to black with brownish elytra (Fig. 44); male tergite VIII truncate and produced apically (Fig. 46); median love of aedeagus and remaining terminalia as illustrated (Figs 45–49)	***Liogluta nitens* (M**ӓ**klin)**
9	Punctures on pronotum and elytra sparse, distance between punctures greater than diameter of a puncture (Fig. 50); surface of disc strongly granulose (Figs 50, 51); male tergite VIII broadly triangularly produced apically and obtusely angular medially (Fig. 53); remaining terminalia and genitalia as illustrated (Figs 52, 54–57)	***Liogluta granulosa* Lohse**
–	Punctures on pronotum and elytra dense, distance between punctures about equal to diameter of a puncture (Figs 58, 65, 72, 79, 86); surface of disc slightly granulose; male tergite VIII truncate apically (Figs 60, 67, 74, 81, 88)	**10**
10	Body length 2.8 mm; pronotum with microsculpture weakly defined (Fig. 86); elytra slightly longer than pronotum (Fig. 86); male tergite VIII truncate apically and evenly serrated, not angulate medially (Fig. 88); median lobe of aedeagus as illustrated (Figs 87); known only from British Columbia	***Liogluta atriventris* (Casey)**
–	Body length 3.9–5.4 mm; pronotum with distinct microsculpture; elytra as long as pronotum or rarely slightly longer (Figs 58, 65, 72, 79); male tergite VIII truncate apically, crenulate or entire, and sometimes angulate medially (Figs 60, 67, 74, 81)	**11**
11	Antennal articles IV-XI yellowish to light brown (Fig. 79); pronotum one-sixth broader than long (Fig. 79); forebody reddish to reddish-yellow (Fig. 79); basal metatarsal article distinctly longer than following article (Fig. 79); male tergite VIII distinctly crenulate apically (Fig. 81); median lobe of aedeagus with apical part of tubus narrow and straight in lateral view (Fig. 80); female sternite VIII emarginate apically (Fig. 84); spermatheca as illustrated (Fig. 85)	***Liogluta intermedia* Klimaszewski & Langor**
–	Antennal articles IV-XI dark brown to black (Figs 58, 65, 72); pronotum usually one-fifth broader than long (Figs 58, 65, 72); forebody dark reddish-brown to almost black (Figs 58, 65, 72); basal metatarsus as long as or slightly longer than following article (Figs 58, 65, 72); male tergite VIII without or with slight crenulation (Figs 60, 67, 74); median lobe of aedeagus with apical part of tubus wide or narrow and arched ventrad in lateral view (Figs 59, 66, 73); female sternite VIII truncate or emarginate medially at apex (Figs 63, 70, 77); spermatheca as illustrated (Figs 64, 71, 78)	**12**
12	Pronotum and elytra dark reddish-brown, without apparent dark brown irregularly shaped spots (Fig. 65); male tergite VIII truncate apically with two lateral teeth (Fig. 67); median lobe of aedeagus with apical part of tubus broad in lateral view (Fig. 66); female sternite VIII with antecostal suture distinctly sinuate and subangulate medially (Fig. 70); spermatheca with very long stem (Fig. 71)	***Liogluta castoris* Klimaszewski & Webster, sp. n.**
–	Pronotum and elytra dark reddish-brown, mottled with dark brown or black irregularly shaped spots (Figs 58, 72); male tergite VIII truncate apically without or with three small apical teeth (Figs 60, 74); median lobe of aedeagus with apical part of tubus narrow in lateral view (Figs 59, 73); female sternite VIII with antecostal suture slightly sinuate (Figs 63, 77); spermatheca with moderately long stem (Figs 64, 78)	**13**
13	Pronotum distinctly transverse (Fig. 72); two basal antennal articles brown (Fig. 72); apical margin of male tergite VIII evenly rounded (Fig. 74); median lobe of aedeagus with apical part of tubus straight in lateral view (Fig. 73); female sternite VIII broadly rounded apically (Fig. 77); spermatheca as illustrated (Fig. 78)	***Liogluta pseudocastoris* Klimaszewski & Webster, sp. n.**
–	Pronotum slightly transverse, appearing subquadrate (Fig. 58); two basal antennal articles reddish-brown (Fig. 58); apical margin of male tergite VIII with subrectangular broad projection with two lateral and one median teeth (Fig. 60); median lobe of aedeagus with apical part of tubus arched ventrad in lateral view (Fig. 59); female sternite VIII emarginate apically (Fig. 63); spermatheca as illustrated (Fig. 64)	***Liogluta microgranulosa* Klimaszewski & Webster, sp. n.**

### *terminalis* species group

This group of species is characterized by: small eyes, with diameter of eye distinctly shorter than postocular area of head in dorsal view (Figs 1, 8, 16, 23, 30); integument of forebody moderately to highly glossy (Figs 1, 8, 16, 23, 30); elytra short, at suture about as long as pronotum (Figs 1, 8, 16, 23, 30); male tergite VIII truncate apically, with or without minute crenulations (Figs 3, 11, 25), except with obtuse triangular projection in *Liogluta quadricollis* (Fig. 18); median lobe of aedeagus with tubus usually arched and moderately narrow apically in lateral view (Figs 2, 9, 10, 17, 24); capsule of spermatheca with apical part compressed, with apical invagination small and short (Figs 7, 15, 22, 29, 34).

#### Liogluta terminalis

Taxon classificationAnimaliaColeopteraStaphylinidae

(Casey, 1906)

Figs 1–7

Anepsiota terminalis Casey, 1906: 339. As Atheta (Liogluta): Bernhauer and Scheerpeltz 1926: 658 (as syn. of Atheta renominata). **Holotype** (female): Canada, British Columbia, Glenora, Wickham; terminalis Casey; Type USNM 39472; Casey bequest 1925; Liogluta terminalis (Casey) V.I. Gusarov 1998; cf. Liogluta aloconoides (USNM). Examined.
Atheta (Liogluta) renominata Bernhauer & Scheerpeltz, 1926: 658 (nom. nov. for Anepsiota terminalis Casey, 1906 in Atheta, not Atheta terminalis Gravenhorst, 1806 and Gyllenhal, 1810; synonymized by Seevers 1978).
Liogluta (Anepsiota) aloconotoides Lohse, in Lohse et al. 1990: 165. **New synonymy. Holotype** (male): Canada, Labrador, L’Anse au Loup, 9.VIII.1972, J.M. Campbell (CNC). **Paratypes**: Canada, Labrador, Red Bay, 8.VIII.1972, J.M. Campbell (5, sex undetermined, CNC).

##### New locality data.

**USA: Montana: Flathead Co.**, Glacier National Park, N Fork Flathead area, 1988, Red Bench Fire study; N Mud Lake, 3520 feet, lodgepole unburned T34N, R21W, Sec 1, 7.VI.1990, pitfall trap, M.A. Ivie (1 ♀, LFC). **New Hampshire**, **Coos Co.**, Hwy. 16, 5–6 km S Gorham, 9.IX.1987, A. Smetana (1 ♂, 2 ♀, CNC).

##### Diagnosis.

This species may be distinguished by the following combination of characters: body subparallel, entirely reddish-brown or with head and posterior abdomen chestnut brown (Fig. 1); length 3.9–4.5 mm; integument of forebody with meshed microsculpture, moderately glossy; head about one-quarter narrower than maximum width of pronotum; pronotum transverse, narrower at base and widest in apical third; elytra at suture about as long as pronotum; basal three articles of metatarsus elongate, first longest, second about as long as third, fourth shorter than either of preceding articles; apical margin of male tergite VIII with broad, short, truncate projection with rounded lateral angles, apical margin smooth or slightly crenulate (Fig. 3); female tergite VIII with apical margin broadly, just visibly emarginate (Fig. 6); genital structures as illustrated (Figs 2, 7).

##### Natural history.

Klimaszewski et al. (2011) reported this species (as *Liogluta aloconotoides*) from various forest types and on coastal limestone barrens in Newfoundland. Specimens from New Brunswick were collected from dung in a coastal red spruce forest, by treading sedges along a small lake margin, from a Lindgren funnel trap deployed in a rich Appalachian hardwood forest with some conifers, and from a pitfall trap (Webster et al. 2012). In Alberta, adults were reared from well-decayed white spruce logs (Klimaszewski et al. 2015). Elsewhere, adults were captured in various forest types including a recently burned forest. The type specimens of *Liogluta aloconotoides* were captured in August (Lohse et al. 1990). Klimaszewski et al. (2015) reported adults from July to October.

##### Distribution.

Recorded from LB, NB, NF, NS, ON, QC, AB, YT, and BC (Casey 1906, Lohse et al. 1990, Klimaszewski et al. 2008, Majka and Klimaszewski 2008, Klimaszewski et al. 2011, Webster et al. 2012, Bousquet et al. 2013, Klimaszewski et al. 2015), and newly in USA from **MT** and **NH**.

##### Comments.

We have examined the female holotype of *Liogluta terminalis* (Casey) from Glenora, British Columbia, and compared it with the specimens of *Liogluta aloconotoides* Lohse east of the Rocky Mountains. We found no external or genital differences between the holotype of *Liogluta terminalis* and the other female specimens identified as *Liogluta aloconotoides* and therefore we consider *Liogluta aloconotoides* as a new synonym of *Liogluta terminalis*.

#### Liogluta trapezicollis

Taxon classificationAnimaliaColeopteraStaphylinidae

Lohse, 1990

Figs 8–15

Liogluta (Anepsiota) trapezicollis Lohse, in Lohse et al. 1990: 165. **Holotype** (male): Canada, **Yukon Territory**, Dempster Hwy., Mi. 60, 3500 ft., 19.VII.1978, J.M. Campbell and A. Smetana (CNC). Not examined.

##### New locality data.

Summarized for 146 specimens captured at 45 collection events from 6 major regions of Southeast Alaska, see http://dx.doi.org/10.7299/X79023ZM for the full data. **USA: Alaska**: Baranof Island (11 specimens, UAM), Chichagof Island (84 specimens, UAM), Dall Island (2 specimens, UAM), Haines, Flower Mountain (3 specimens, UAM), Hawthorne Peak (45 specimens, UAM), South Chilkat Peninsula (1 specimen, UAM). Excel file with locality data is available from LFC.

##### Diagnosis.

This species may be distinguished by the following combination of characters: body subparallel, slender, dark brown to black with pronotum brown and paler than head, elytra yellowish or reddish-brown (Fig. 8); length 3.8–4.4 mm; integument of forebody with meshed microsculpture moderately pronounced, surface moderately glossy; head about one-quarter narrower than maximum width of pronotum; pronotum transverse, narrower at base and widest at middle (width of pronotum variable, some specimens have pronotum markedly narrower than base of elytra and some have pronotum nearly as wide as base of elytra); elytra at suture slightly shorter than pronotum; basal three articles of metatarsus elongate, subequal in length and each slightly longer than fourth article; male tergite VIII with apical margin truncate, bordered by two short lateral teeth, variably sculptured and ranging from smooth to crenulate, or denticulate along margin (Fig. 11); genital structures as illustrated (Figs 9–15).

##### Natural history.

The holotype was collected in July (Lohse et al. 1990). The Alaskan specimens were collected in July only from alpine zones between 453 and 1071 m elevation, none were collected in lowland forests. Habitats include alpine flood meadows, under rocks, herbaceous heath with *Luetkea*, *Cassiopes*, and *Lupinus*, low rocky tundra with *Dryas*, meadow heath with *Phyllodoce*, *Senecio*, and *Luetkea*, shrubby krummholz with *Elliottia* and *Tsuga*, wet meadows with *Carex*, *Petasites*, *Senecio* and *Ranunculus*.

##### Distribution.

Canada: **BC**, YT (Klimaszewski et al. 2012). USA: **AK** (Lohse et al. 1990).

##### Comments.

We were not able to compare types of *Liogluta trapezicollis* Lohse with the specimens we examined, and our determinations are based on the published description by Lohse in Lohse et al. (1990). The types of *Liogluta trapezicollis* housed in the Canadian National Collection of Insects were borrowed several years ago by V. Gusarov (Oslo, Norway) and our persistent efforts to have these specimens returned to Canada have failed.

Five specimens in UAM were successfully DNA barcoded (UAM GUID, BOLD Process ID): UAM:Ento:145576, UAMIC2696–15; UAM:Ento:145623, UAMIC2740–15; UAM:Ento:152467, UAMIC2750–15; UAM:Ento:232527, UAMIC2677–15; UAM:Ento:232696, UAMIC2753–15. The DNA sequences for these specimens are all very similar (maximum distance of 0.32%, nearest neighbour of 3.13%) and fall within the same BIN (Barcode Index Number) (Ratnasingham and Hebert 2013), BOLD:ACU9772, which is not shared by any other species.

#### Liogluta quadricollis

Taxon classificationAnimaliaColeopteraStaphylinidae

(Casey, 1894)

Figs 16–22

Anepsiota quadricollis Casey, 1894: 330. As Atheta (Liogluta): Bernhauer and Scheerpeltz 1926: 658. **Holotype** (female). Canada, **British Columbia**, Vancouver Island, Anepsiota quadricollis; Type USNM 39471 (USNM). Examined.

##### New locality data.

Canada: **British Columbia**: Hwy 5 at Juliet Creek, 25.IX.1994, Lot 2 [in forest under rocks in poplar stand], B.F. & J.L. Carr, J. & B. Carr Coll., bequested to CNC August 2000 (1 ♂, CNC).

##### Diagnosis.

This species may be distinguished by the following combination of characters: body subparallel, slender, bicoloured, pronotum orange and remainder of body dark brown to reddish-brown; length 4.1–4.3 mm (Fig. 16); integument of forebody with weak meshed microsculpture, surface highly glossy; head slightly narrower than pronotum; pronotum subquadrate; antennae enlarged and black to brown; elytra about as wide as pronotum and at suture about as long as pronotum; basal two articles of metatarsus distinctly elongate, subequal in length, each longer than third article. **New description of male.** Apical margin of tergite VIII with broad, obtusely triangular projection in almost middle half with rounded lateral angles (Fig. 18); sternite VIII elongate, rounded apically, with antecostal suture arcuate, well separated from basal margin (Fig. 19); median lobe of aedeagus with tubus short and sinuate, its subapical part narrowly elongate in lateral view, internal sac structures distinct (Fig. 17). **Female.** Tergite VIII with apical margin obtusely angulate, broadly rounded at middle (Fig. 20); sternite VIII shallowly, broadly emarginated medially (Fig. 21); spermatheca with a short club-shaped capsule and short apical invagination, stem long and highly sinuate (Fig. 22).

##### Natural history.

The holotype and the other BC specimen were collected in September, the Carrs collected a male under a rock in a poplar stand.

##### Distribution.

Vancouver Island, British Columbia (Casey 1894).

#### Liogluta wickhami

Taxon classificationAnimaliaColeopteraStaphylinidae

(Casey, 1894)

Figs 23–29

Anepsiota wickhami Casey, 1894: 331. As Atheta (Liogluta): Bernhauer and Scheerpeltz 1926: 656. **Holotype** (female): Canada, **British Columbia**; Stickeen River Canyon; Anepsiota wickhami; Type USNM 39474, Casey bequest 1925 (USNM). Examined.

##### New locality data.

Canada: **British Columbia**: Mi. 56 Haines Hwy., Three Guardsmen Pass, 4.VII.1968, 3200 feet, J.M. Campbell and A. Smetana (1 ♂, CNC).

##### Diagnosis.

This species may be distinguished by the following combination of characters: body broadly subparallel (Fig. 23); pronotum, elytra, legs and basal antennal article reddish-brown, head and abdomen chestnut brown (Fig. 23); length 4.0–4.2 mm; integument of forebody with meshed microsculpture, moderately glossy; head about one-third narrower than maximum width of pronotum; pronotum more or less evenly arcuate laterally (Fig. 23); elytra at suture about as long as pronotum; basal three articles of metatarsus missing in holotype (Fig. 23). **New description of male.** Apical margin of tergite VIII with very broad, short, subtruncate projection with rounded lateral angles, with apical margin faintly crenulate (Fig. 25); sternite VIII broadly rounded apically (Fig. 26); median lobe of aedeagus with tubus slightly arcuate ventrally, with apex narrow and rounded (Fig. 24). **Female.** Tergite VIII truncate apically (Fig. 27); sternite VIII very slightly broadly emarginate apically (Fig. 28); spermatheca with tubular capsule and deep apical invagination, stem thin, long, and highly sinuate (Fig. 29).

This species is similar to *Liogluta terminalis* but has dark brown antennae, head and pronotum (antennae, head, and pronotum are uniformly reddish-brown or only slightly darker than remaining parts of the body in *Liogluta terminalis*). Spermatheca is differently shaped in each species; *Liogluta wickhami* has smaller and differently shaped capsule with a deep apical invagination and has a shorter and differently looped posterior stem (Fig. 29). *Liogluta wickhami* is also very similar to *Liogluta vasta* but can be distinguished by the shape of pronotum which has evenly arcuate sides and is broadest at middle (Fig. 23), while it is trapezoidal in shape and is broadest in apical third in the latter species (Fig. 30).

##### Natural history.

Unknown.

##### Distribution.

The female holotype was captured in the Stickeen River Valley of British Columbia (Casey 1894), and one male was found in Three Guardsmen Pass, British Columbia.

#### Liogluta vasta

Taxon classificationAnimaliaColeopteraStaphylinidae

(Mӓklin, 1853)

Figs 30–34

Homalota vasta Mӓklin, 1853: 183. As Atheta (Liogluta): Moore and Legner 1975: 376; as Liogluta: Lohse and Smetana 1985: 297, Gusarov 2003a: 39. **Lectotype** (female): USA, **Alaska**: Sitka; Holmberg; Mus. Zool. H:fors, Spec. Typ. No. 2251, Homalota vasta Mӓkl.; Mus. Zool. Helsinki, Loan No. C98–138; Paralectotypus Homalota vasta Mӓklin, Lohse des. 1985, Gusarov rev. 2000; http://id.luomus.fi/GAC. 16963, UNITED STATES **Alaska**, Sitka, 57.1483N, 135.23W, Holmberg leg. Examined. We have added a new lectotype label (see discussion below) [there was no original label designated by Lohse]. **Paralectotype** (female): USA, **Alaska**: Sitka; Holmberg; Homalota vasta m. Sitkcha [Sitka]; Mus. Zool. Typ. No. 2250, Homalota vasta Mӓkl.; typus; Mus. Zool. Helsinki, Loan No. C 14527; Mus. Zool. Helsinki, Loan No. C 98; Lectotypus Homalota vasta Mӓklin, Lohse des. 1985, Gusarov rev. 2000; http://id.luomus.fi/GAC. 16962, UNITED STATES **Alaska**, Sitka, 57.1483N, 135.23W, Holmberg leg. Examined. We have added a new Paralectotype label (see discussion below) [there was no original label designated by Lohse].

##### Diagnosis

(based on female lectotype, male unknown). This species is very similar to *Liogluta wickhami*, but in *Liogluta vasta* the forebody is less reddish, and the pronotum is more trapezoidal and narrowest at base (Fig. 30); the apical margin of tergite VIII is broadly arcuate with the antecostal suture very narrowly separated from the basal margin (Fig. 32); the apical margin of sternite VIII is slightly, very broadly emarginate with the antecostal suture highly sinuate and well separated from the basal margin (Fig. 33).

The female lectotype is missing the spermatheca. The spermatheca of the Yukon specimen in CNC cited by Lohse and Smetana (1985), tentatively identified as belonging to this species, is illustrated in Fig. 34 (after Lohse and Smetana 1985). Males and more females from the type locality are needed to clearly define this species, which is here tentatively listed as a valid species. When more specimens of *Liogluta vasta* become available for study and the morphological variation is known, we will be able to understand the relationship of this species to other nearctic *Liogluta* species. *Liogluta vasta* is also similar to some specimens (with broad pronotum) of *Liogluta trapezicollis*. A DNA comparison between *Liogluta vasta* and other *Liogluta* species is needed to clarify its identity and relationships.

##### Distribution.

Canada: YT?. USA: AK.

##### Discussion.

The original type material of *Homalota vasta* Mӓklin, 1853 (ZMH) consists of two female specimens representing two different species in two genera, *Atheta* (as *Boreophilia* in Lohse and Smetana 1985) and *Liogluta*. Lohse and Smetana (1985) designated the female specimen belonging to *Liogluta* as the lectotype of *Homalota vasta* Mӓklin. However, the label data published by Lohse and Smetana (1985) for the *Liogluta* specimen corresponded to the *Atheta* specimen (see also discussion in Gusarov 2003b, who also mislabelled the specimens). We consider Lohse and Smetana’s lectotype designation as valid regardless of the obvious mistake of publishing the wrong label data; therefore, the name *vasta* is affiliated with *Liogluta*. The female paralectotype belongs to *Atheta keeni* Casey, 1910.

It is noteworthy that despite years of intensive collections made primarily between 2008–2013 in southeast Alaskan lowland forests and alpine zones, including in and around Sitka, which have resulted in 22,029 specimens of Staphylinidae (http://arctos.database.museum/saved/SE-AK-Staphylinidae), no specimens of *Liogluta vasta* were found.

### *nigropolita* species group

This group of species has a body shape non-typical for *Liogluta* and it resembles some of the Atheta (Dimetrota) species with elytra distinctly wider than head and pronotum (Figs 35, 44). This group is characterized by large and bulging eyes, diameter of eye about as long as postocular area of head in dorsal view (Figs 35, 44); integument of forebody highly glossy (Figs 35, 44); elytra at suture at least as long as pronotum (Figs 35, 44), and elytra about one-third broader than pronotum (Figs 35, 44); male tergite VIII truncate apically and apical margin entire or slightly produced with rounded lateral teeth (Figs 38, 46); tubus of the median lobe of the aedeagus is arched, or almost straight and in lateral view moderately narrow apically (Figs 36, 45); spermatheca with spherical capsule with moderately long apical invagination and sinuate stem (Figs 42, 43). The spermatheca of *Liogluta nitens* was not found and may be very small, not sclerotized, or absent.

#### Liogluta nigropolita

Taxon classificationAnimaliaColeopteraStaphylinidae

(Bernhauer, 1907)

Figs 35–43

Atheta nigropolita Bernhauer, 1907: 390. As Liogluta: Lohse and Smetana 1985: 286.
Liogluta nigropolita **Syntype** (male): USA, **New Hampshire**, Mt. Washington (FMNH).

##### New locality data.

CANADA: **Quebec**: Gt. Whale Riv., 5.IX.1949, J.R. Vockeroth (1 sex undetermined, CNC). **Northwest Territories**: Yellowknife, 62.50714°N, 113.39443°W, 236 m, mesic habitat replicate #2, Yellow Pan Trap #2, 15–18.VI.2011, col. NBP Field Party (1 ♀, LFC).

##### Diagnosis.

This species may be distinguished by the following combination of characters: body elongate with elytra and abdomen broad, moderately robust, dark brown to black with tarsi and tibiae often reddish-brown, elytra sometimes with reddish tinge (Figs 35); length 3.5–4.0 mm; integument of forebody with moderately pronounced meshed microsculpture, surface highly glossy; head about one-eighth narrower than maximum width of pronotum (Fig. 35); pronotum transverse, about evenly wide in posterior half, then distinctly narrowing apicad, forming round apical angles (Fig. 35); elytra at suture slightly longer than pronotum (Fig. 35); basal four articles of metatarsus about the same length, each shorter than fifth article. **Male.** Tergite VIII with apical margin broadly arcuate, without teeth or crenulations (Fig. 38); sternite VIII rounded apically (Fig. 39); median lobe of aedeagus with tubus broadly arcuate ventrally and with apex narrow and pointed in lateral view (Fig. 36); tubus broad and triangular apically in dorsal view (Fig. 37). **Female.** Tergite VIII broadly arcuate apically (Fig. 40); sternite VIII evenly rounded apically, with antecostal suture distinctly sinuate (Fig. 41); spermatheca with spherical capsule with invagination deep and narrow, stem S-shaped, gradually becoming very narrow posteriad (Figs 42, 43).

##### Natural history.

Adults occur in moss and leaf litter (Lohse et al. 1990).

##### Distribution.

Canada: LB, NF, **NT**, NU, QC, YT. USA: NH (Lohse et al. 1990; Klimaszewski et al. 2012; Bousquet et al. 2013).

##### Comments.

This species is probably transcontinental in northern Canada.

#### Liogluta nitens

Taxon classificationAnimaliaColeopteraStaphylinidae

(Mӓklin, 1852)

Figs 44–49

Homalota nitens Mӓklin, 1852: 307. As Liogluta: Lohse and Smetana 1985: 288.
Liogluta nitens (male): USA, **Alaska**: Sitcha [Sitka], Holmberg, coll Mӓklin; Homalota nitens Mkln., Sitka pr. Hlm. Berg (ZMH). Designated by Lohse and Smetana (1985).
Liogluta apposita (Casey, 1911). Synonymized by Gusarov 2003b [type locality BC: Metlakatla].
Liogluta insolens (Casey, 1910). Synonymized by Gusarov 2003b [type locality BC: Queen Charlotte Islands: Massett].
Liogluta resplendens (Casey, 1910). Synonymized by Gusarov 2003b [type locality: BC: Queen Charlotte Islands].

##### New locality data.

CANADA: **Alberta**: 28 km NW Hinton, 0.5 km S of Rock Lake Road, 53.524°N, 117.957°W, Ecosite Surrogacy Study, Ecoregion: UF, Ecosite C2, Stand C205, pitfall trap 2, 5, 14.V-4.VI.2004, J. Hammond et al. (1 ♂, 9 ♀, NoFC); same data except: 30.VII-13.VIII.2004, pitfall trap 5 (3 ♂, 2 ♀, NoFC); 23.1 km NW Hinton, W.A. Switzer Prov. Pk., 53.560°N, 117.808°W, Ecosite Surrogacy Study, Ecoregion: UF, Ecosite F2, Stand F214, pitfall trap 5, 31.VII-13.VIII.2004, J. Hammond et al. (1 ♂, NoFC); same data except: Stand F216, pitfall trap 4 (1 ♀, NoFC); 32 km NW Hinton, 0.5 km E Wild Hay Campgr., 53.529°N, 117.946°W, Ecosite Surrogacy Study, Ecoregion: UF, Ecosite F2, Stand F216, pitfall trap 3, 16–30.VII.2004, J. Hammond et al. (1 ♂, 1 ♀, NoFC); same data except: pitfall trap 4, 30.VII-13.VIII.2004 (1 ♂, NoFC); 57 km N Hinton, 1.5 km W of J. Wright Rd., 53.921°N, 117.617°W, Ecosite F1, Stand F101, pitfall trap 2, 28.VII-11.VIII.204, J. Hammond et al. (2 ♀, NoFC); 62 km N Hinton, 5 km W of J. Wright Rd., 53.969°N, 117.668°W, Stand F105, 30.VI-14.VII.2004, J. Hammond et al. (1 ♀, NoFC); same data except: 53.921°N, 117.663°W, Stand F202, pitfall trap 1, 28.VII-11.VIII.2004, J. Hammond et al. (1 ♂, NoFC).

##### Diagnosis.

This species may be distinguished by the following combination of characters: body narrowly elongate, robust, dark-brown to black with elytra, tarsi and tibiae often yellowish- or reddish-brown (Fig. 44); length 2.8–3.3 mm; integument of forebody with moderately pronounced meshed microsculpture, surface highly glossy (Fig. 44); head about one-eighth narrower than maximum width of pronotum (Fig. 44); pronotum transverse, about evenly wide in basal half and then distinctly narrowing anteriad (Fig. 44); elytra at suture about as long as pronotum (Fig. 44); basal four articles of metatarsus about same length, each shorter than fifth article. **Male.** Tergite VIII with short subrectangular projection on more than half width of apical margin, with rounded lateral angles, apical margin smooth or micro-crenulate (Fig. 46); sternite VIII parabolically rounded apically (Fig. 47); median lobe of aedeagus with tubus almost straight in lateral view, with apex moderately narrow, rounded (Fig. 45). **Female.** Tergite VIII truncate apically (Fig. 48); sternite VIII with apical margin evenly rounded, antecostal suture slightly sinuate (Fig. 49). Spermatheca unknown.

##### Natural history.

Adults were captured using pitfall traps in Carmanah Valley, Vancouver Island, from June to September, with the peak catch in June (Klimaszewski and Winchester 2002). They were found mainly in the interior and transition zones of a Sitka spruce forest (Klimaszewski and Winchester 2002). Several adults were collected from moss at the edge of an old road in the Queen Charlotte Islands, British Columbia. In Alberta, adults were collected in pitfall traps in various forest types in the Upper Cordilleran Ecoregion. Adults in Alaska were collected in a wide variety of habitats spanning lowland forests to alpine zones: alpine meadow litter, lowland forest clearcuts, floodplain meadows with *Athyrium*, *Caltha*, and *Rubus*, under rocks, in krummholz alpine habitats of *Tsuga mertensiana*, near bear dung in alpine habitats, old growth temperate rain coniferous forests, alpine heath with *Empetrum*, and *Vaccinium*, subalpine habitats with *Salix*, and *Veratrum*.

##### Distribution.

Canada: **AB**, BC, YT. USA: AK, OR, WA (Mӓklin 1852, Bernhauer 1907, Hatch 1957, Moore and Legner 1975, Lohse and Smetana 1985, Klimaszewski and Winchester 2002).

##### Comments.

There is considerable variation in length and width of elytra in specimens from Vancouver Island, Oregon (having broader and longer elytra), and those with narrow and shorter elytra from the Queen Charlotte Islands, Alberta, and Alaska. The genitalic features were the same in those of the typical form with the longer and broader elytra, and those with narrower and shorter elytra. Therefore, we consider this as intraspecific variation. Additional studies, including DNA comparison, are needed to reveal the relationship between these two morphotypes. Two UAM Alaskan specimens (UAM:Ento:152502, UAM:Ento:232546) were DNA barcoded (UAMIC2665–15, UAMIC2701–15) and they cluster closely with two specimens of this species DNA barcoded from Alberta, Canada.

### *granulosa* species group

This group of species is characterized by: body medium-sized and subparallel (Fig. 50), eyes large and bulging, diameter of eye about as long as postocular area of head in dorsal view (Fig. 50); integument of forebody highly glossy (Fig. 50); elytra about one-fifth broader than pronotum, at suture about as long as pronotum (Fig. 50); elytra sparsely and strongly granulose (Fig. 51); apical margin of male tergite VIII with broad, short obtusely angular projection medially (Fig. 53); median lobe of aedeagus with tubus slightly arched ventrad, moderately narrow apically in lateral view (Fig. 52).

#### Liogluta granulosa

Taxon classificationAnimaliaColeopteraStaphylinidae

Lohse, 1990

Figs 50–57

Liogluta (Liogluta) granulosa Lohse, in Lohse et al. 1990: 164. **Holotype** (male): USA, **Alaska**, King Salmon, Naknek R. Alaska, 6.VII.1952, W.R. Mason, No. 20313 (CNC). Examined.

##### New locality data.

CANADA: **Yukon Territory**: location EMAN Plot, Cadet Camp, EP-Yukon, 15.X.2001 (1 ♀, NoFC); Tombstone Mts., 64.60560°, 138.36413°, Rep. 1, mesic, yellow pan trap, 21–24.VI.2011, NBP Field Party (1 ♀, LFC).

USA: **Alaska**: Quinhagak site G, 3 m elevation, 59.71035°, 161.89102°, dry tundra, between *Rubus* sp. hummocks, pitfall, 18–26, VIII.2014, V. Forbes (1 ♂, LFC) [submitted for barcoding]; Naknek, 58.73973°N -157.0636°W, 2–5 m elev., creekside/ocean beach confluence, under boards and driftwood 10.VI.2007, D.S. Sikes. UAM:Ento:29798 (1 ♂, UAM) [DNA barcoded: http://arctos.database.museum/guid/UAM:Ento:29798].

##### Diagnosis.

This species may be distinguished by the following combination of characters: body broadly subparallel, dark brown, with elytra, tarsi and tibiae often reddish-brown (Fig. 50) (one specimen from northern Yukon was entirely black); length 2.8–3.3 mm; integument of forebody with moderately pronounced meshed microsculpture; head about one-eighth narrower than maximum width of pronotum (Fig. 50); pronotum transverse, about evenly wide in basal one-third of its length, then strongly broadest at apical one-third and gradually narrowed apically (Fig. 50); elytra at suture about as long as pronotum, its surface coarsely granulose (Fig. 50); basal two articles of metatarsus about the same length, each shorter than fifth article. **Male.** Apical margin of tergite VIII with short, very obtusely angular projection in medial two-thirds with rounded lateral angles, margin of projection smooth or micro-denticulate (Fig. 53); apical margin of sternite VIII broadly parabolic (Fig. 54); median lobe of aedeagus with tubus broadly arched, bent ventrad, apex narrow and rounded (Fig. 52). **Female.** Apical margin of tergite VIII truncate in middle one-third (Fig. 55); apical margin of sternite VIII arcuate, antecostal suture distinctly sinuate (Fig. 56); spermatheca highly sinuate as illustrated (Fig. 57).

##### Natural history.

Adults were captured in June, July, August, and October. One Alaskan specimen was captured in tundra between *Rubus* species and another at a creekside/ocean beach confluence, under boards and driftwood.

##### Distribution.

Canada: **YT**. USA: AK (Lohse et al. 1990, Klimaszewski et al. 2008, Klimaszewski et al. 2012).

##### Comments.

Only a few specimens of this species are known. Its distribution is nordic and the habitat is unknown. One specimen (UAM:Ento:29798) in UAM was DNA barcoded (UAMIC2693–15), the first and only for this species so far.

### *microgranulosa* species group

This group of species is characterized by: body medium- to large-sized (length 2.8–5.4 mm), subparallel, eyes large and bulging, diameter of eye about as long as postocular area of head in dorsal view (Figs 58, 65, 72, 79, 86); integument of forebody moderately glossy (Figs 58, 65, 72, 79, 86); elytra about 20–24% broader than pronotum, at suture at least as long as pronotum (Figs 58, 65, 72, 79, 86); elytra densely and finely granulose in most species (Figs 58, 65, 72, 79); apical margin of male tergite VIII usually with well-developed broad projection, with margin straight, entire, serrate or obtusely angulate (Figs 60, 67, 81, 88) or apical margin very obtusely angulate in middle; median lobe of aedeagus with tubus straight to distinctly arched ventrad and narrow to moderately wide apically in lateral view (Figs 59, 66, 73, 80, 87); spermatheca basically S-shaped, capsule with invagination short or deep (Figs 64, 71, 78, 85).

#### Liogluta microgranulosa

Taxon classificationAnimaliaColeopteraStaphylinidae

Klimaszewski & Webster sp. n.

http://zoobank.org/A0ED06AF-6A25-43A0-9B70-D714A3252642

Figs 58–64

##### Holotype

**(male). Canada**, **New Brunswick**, Restigouche Co., Jacquet River Gorge P.N.A., 47.7361°N, 66.0778°W, 16.VIII.2010, R.P. Webster // beaver dam, among sticks and debris near an overflow area of dam (near flowing water) (LFC). **Paratypes**: same data as holotype (1 ♂, 3 ♀, LFC; 2 ♂, 7 ♀, NBM; 4 ♂, 5 ♀, 1 sex undetermined, RWC); Jacquet River Gorge P.N.A., 47.7357°N, 66.0774°W, 24.VII.2008, R.P. Webster// Margin of pond, among leaves and sedges near pond margin (1♀, LFC). York Co., Fredericton, 45.9361°N, 66.6747°W, 17.VIII.2009, R.P. Webster // Beaver dam, outer margin under overhanging sticks near water (1 ♀, RWC).

##### Etymology.

*Microgranulosa* is a Latin adjective meaning microgranulate, in reference to the minute sculpture on the elytra of this species.

##### Description.

This species may be distinguished by the following combination of characters: body narrowly subparallel; head, apical articles of antennae, and posterior part of abdomen black, elytra brownish and mottled with black, remaining parts reddish-brown (Fig. 58); length 4.6–5.1 mm; integument of forebody with moderately pronounced meshed microsculpture, surface moderately glossy (Fig. 58); head about one-quarter narrower than maximum width of pronotum (Fig. 58); pronotum transverse, about evenly wide in basal half of its length, then strongly narrowed apically (Fig. 58); elytra at suture about as long as pronotum, surface finely and densely microgranulose; basal three articles of metatarsus about equally elongate, each longer than fourth article. **Male.** Apical margin of tergite VIII with very broad, very obtusely angular projection, with obtuse lateral angles and small tooth medially, margin often micro-crenulate (Fig. 60); sternite VIII rounded apically (Fig. 61); median lobe of aedeagus with tubus distinctly arched ventrad in apical half, apical part narrow (Fig. 59). **Female.** Tergite VIII with apical margin obtusely angulate (Fig. 62); sternite VIII with apical margin slightly emarginate medially (Fig. 63); spermatheca with stem long, sinuate, spiral posteriorly, capsule club-shaped with apical invagination deep and narrow (Fig. 64).

##### Distribution.

Canada: Known only from New Brunswick, Canada.

##### Natural history.

Nearly all adults from New Brunswick were collected from American beaver (*Castor canadensis* Kuhl) dams. Most were collected from among sticks and debris near an overflow area of the dam, another from under overhanging sticks on the outer margin of the dam. One individual was collected from among leaves and sedges near a pond margin. Specimens were collected in July and August.

#### Liogluta castoris

Taxon classificationAnimaliaColeopteraStaphylinidae

Klimaszewski & Webster sp. n.

http://zoobank.org/5BCB34A3-CFE7-4230-A638-83FB11AC6D89

Figs 65–71

##### Holotype

(male). **Canada**, **New Brunswick**, York Co., Charters Settlement, 45.8395°N, 66.7391°W, 21.IV.2010, R.P. Webster coll. //Mixed forest opening, collected with net during evening flight between 16:30 and 19:00 h (LFC). **Paratypes.** York Co., same data as holotype except (2 ♀, RWC); same data as holotype except: 17.VI.2005 // mixed forest in flight (1 ♂, LFC) [barcoded BIO]; same data as holotype except 23.IV.2008 // Mixed forest, in flight, collected with net between 15:00 and 18:00 h (1 ♂, RWC); same data as holotype except 5.IV.2010 // Mixed forest opening, collected with net during evening flight between 16:30 and 19:00 h (1 ♂, RWC); Charters Settlement, 45.8456°N, 66.7267°W, 5.V.2010, 16.V.2010, beaver dam, among sticks and debris near overflow area of dam, near flowing water (1 ♀, LFC; 2 ♂, 1 ♀, RWC); Charters Settlement, 45.8331°N, 66.7279°W, 20.V.2010, among sticks and debris near overflow area of dam, near flowing water (1 ♂, RWC). Saint John Co., ca 2 km NE of Maces Bay, 45.1161°N, 66.4560°W, 8.V.2006, R.P. Webster, eastern white cedar swamp, in sphagnum and litter near brook (1 ♀, RWC). **Nova Scotia**: Cape Breton H.N.P., North Mtn., 15.VIII.1983, J.E.H. & R.J. Martin (1 ♂, CNC); Cape Breton H.N.P., Lone Shieling, PG729861, 19.VI.1983, Y. Bousquet, interception trap (1 ♂, CNC); Cape Breton H.N.P., Lone Shieling, PG729861, 3.VI.1983, H. Goulet, Pans, Malaise (1 ♂, CNC). **Québec**: Gatineau Pk., near Mud Lake, 24.X.1967, A. Smetana (2 ♂, CNC).

##### Etymology.

*Castoris* is a Latin adjective derived from the name of the American beaver (*Castor canadensis* Kuhl), in reference to beaver dams where some of the type specimens were captured.

##### Description.

Body length 4.6–5.4 mm, subparallel (Fig. 65); head and at least apical part of abdomen dark brown with pronotum, elytra, basal articles of antennae and legs yellowish to reddish-brown; integument moderately glossy, more so on posterior abdomen; forebody with minute and sparse punctation and sparse pubescence (Fig. 65); elytra with minute micro-granulation; head rounded and narrowed posteriorly, with large eyes, each about as long as postocular area in dorsal view (Fig. 65); antennae with articles V-X subquadrate to slightly elongate (Fig. 65); pronotum slightly transverse, broadly rounded laterally, slightly wider than head and narrower than elytra, pubescence directed latero-posteriad from midline of disc (Fig. 65); elytra transverse, at suture as long as pronotum, slightly longer laterally, with pubescence directed posteriad (Fig. 65); abdomen subparallel for most of its length, about as wide as elytra (Fig. 65). **Male.** Aedeagus with bulbus narrowly oval, median lobe with apical half of tubus slightly arched ventrad, apical part moderately broad in lateral view (Fig. 66); internal sac with few pronounced structures/membrane folds (Fig. 66); apical margin of tergite VIII with very broad truncate projection with obtuse lateral angles, with margin smooth or minutely crenulate (Fig. 67); apical margin of sternite VIII rounded (Fig. 68). **Female.** Tergite VIII with apical margin broadly rounded (Fig. 69); sternite VIII scarcely emarginate apically, antecostal suture distinctly sinuate, well separated from basal margin (Fig. 70); spermatheca with stem long, sinuate, twisted posteriorly, capsule tubular, with apical invagination narrow, short (Fig. 71).

##### Natural history.

In New Brunswick, adults were collected using an aerial (butterfly) net in a mixed forest opening during evening flights (between 15:00 and 19:00 h) during April and May. A number of individuals were collected from among sticks and debris near the overflow area of a beaver dam during May. One individual was sifted from sphagnum and litter near a brook in an eastern white cedar swamp in May. In Nova Scotia, specimens were captured in flight interception, pan, and Malaise traps during the months of June and August. The single specimen from Ontario was captured in October.

##### Distribution.

Canada: Known from NB, NS, QC.

##### Comments.

This species is similar to *Liogluta microgranulosa* but in *Liogluta castoris* the pronotum and elytra are more elongate and more reddish-brown (Fig. 65); the median lobe of the aedeagus has the apical part of the tubus broader and shorter in lateral view (Fig. 66); male tergite VIII is truncate and not at all angulate medially (Fig. 67); the spermatheca has a longer stem (Fig. 71); and female sternite VIII has an apical emargination which is much less noticeable and the antecostal suture is more distinctly sinuate (Fig. 70).

#### Liogluta pseudocastoris

Taxon classificationAnimaliaColeopteraStaphylinidae

Klimaszewski & Webster sp. n.

http://zoobank.org/D8CBE451-DB4D-47DA-8B69-59A39C2D381C

Figs 72–78

##### Holotype

(male). **Canada**, **New Brunswick**, York Co., Charters Settlement, 45.8456°N, 66.7267°W, 10.VI.2010, R.P. Webster, coll., beaver dam among sticks and debris near an overflow area of dam, near flowing water (LFC). **Paratypes.** same data as holotype: (2 ♂, 1 ♀, RWC): same data as holotype except 16.V.2010 (2 ♀, RWC); **New Brunswick: York Co.**, Charters Settlement, 45.8395°N, 66.7391°W, 3.V.2012, R.P. Webster, mixed forest opening, during evening flight between 16:30 and 19:00 h (1 ♂, LFC [barcoded BIO]; 1 ♀, RWC).

##### Etymology.

*Pseudocastoris* is the Latin prefix *pseudo*-, false, added to the species name *castoris*, reflecting the close similarity of the two species.

##### Description.

Body length 3.9–4.4 mm, subparallel; dark brown with irregularly shaped lighter areas on pronotum in some individuals, head and abdomen dark brown, antennae dark, and legs yellowish; integument moderately glossy, more so on posterior portion of abdomen; forebody with minute and sparse punctation and sparse pubescence (Fig. 72); elytra with micro-granulation (Fig. 72); head rounded and narrowed posteriorly, eyes large, each about as long as postocular area in dorsal view (Fig. 72); antennae with articles V-X subquadrate to slightly transverse (Fig. 72); pronotum transverse, broadly rounded laterally, slightly wider than head and narrower than elytra, pubescence directed latero-posteriad from midline of disc (Fig. 72); elytra transverse, at suture about as long as pronotum, slightly longer laterally, with pubescence directed posteriad; abdomen subparallel for most of its length, about as wide as elytra (Fig. 72). **Male.** Tergite VIII broadly rounded apically, margin smooth (Fig. 74); apical margin of sternite VIII broadly parabolic (Fig. 75); median lobe of aedeagus with bulbus narrowly oval, tubus almost straight with apical part narrowly rounded in lateral view (Fig. 73); internal sac without distinct sclerites but with some vaguely-shaped structures (Fig. 73). **Female.** Tergite VIII broadly rounded apically (Fig. 76); sternite VIII a little less broadly rounded apically, antecostal suture slightly sinuate, moderately separated from basal margin (Fig. 77); spermatheca with capsule club-shaped, [invagination not perceptible], stem sinuate, about equally narrow throughout with only posterior part enlarged but not twisted (Fig. 78).

##### Natural history.

Most individuals were collected from among sticks and debris near an overflow area of a beaver dam during May and June. Others were collected using an aerial (butterfly) net in a mixed forest opening during an evening flight (between 16:30 and 19:00 h) during May.

##### Distribution.

Known only from New Brunswick, Canada.

##### Comments.

This species is closely related to *Liogluta castoris* and *Liogluta microgranulosa* but in *Liogluta pseudocastoris* the body is darker, particularly the pronotum, the pronotum is strongly narrowed basally with more angular posterior angles (Fig. 72); the shape of the median lobe of the aedeagus is different in lateral view, with the apical part narrower and very slightly arched ventrad (Fig. 73); the apical margin of male tergite VIII is evenly rounded (Fig. 74); the apical margin of female sternite VIII is not emarginate, with the antecostal suture only slightly sinuate (Fig. 77), and the shape of the spermatheca is different, with the posterior part of the stem enlarged but not twisted (Fig. 78).

#### Liogluta intermedia

Taxon classificationAnimaliaColeopteraStaphylinidae

Klimaszewski & Langor

Figs 79–85

Liogluta intermedia Klimaszewski & Langor, 2011: 168. **Holotype** (female): Canada, Newfoundland, Baie Verte Pen., 10 km SE Pumbly Cove, 49.68°N, 56.62°W, 3.X.2006, Site D, ex pitfall trap in riparian forest// NL Dept. Env. & Conserv., Riparian Biodiversity Study, Site D Trap C5, (LFC).

##### New locality data.

CANADA: **Newfoundland**: Notre Dame Jct. Prov. Pk., 49.116°N, 55.079°W, pitfall trap, conifer forest, 27.VIII.2011, col. L. Pollett (1 ♂, LFC); same data except: 20.VIII.2011 (1 ♂, LFC); 13.IX.2011 (1 ♂, 1 ♀, 1 sex undetermined, LFC). **S-W Labrador**: 40 km W Churchill Falls, Rt. 500, km 229, 53.373°N, 64.309°W, 12–26.VIII.2001, S. & J. Peck, carrion trap, elevation 550 m, Spruce-moss forest (1 ♀, LFC). **Nova Scotia**: Cape Breton H.N.P., Lone Shieling, 60 m, PG730860, 15.IX.1984, J.M. Campbell & A. Davies, sifting litter and moss (1 ♂, 3 ♀, CNC); Cape Breton H.N.P., 5 m, S. Ingonish Harbour, PG963674, 12.IX.1984, J.M. Campbell & A. Davies, tread flooded Carex and grasses (1 ♀, CNC); **Hants Co.**, Upper Rawadon, 21.VII.2009, J. Renkema, highbush blueberry field R3T5C (1 sex undetermined, LFC); same data except: 25.VI.2009, highbush blueberry field R2T4A (1 ♀, LFC). **Québec**: Scotstown, 15.V.2006, 2.X.2006, 9.X.2006, 22.X.2006, 23.X.2006, C. Levesque (4 ♂, 2 ♀, LFC; Mt. Orford Pk., 20.IX.-11.X.1972, Dondale & Redner (1 ♀, CNC); Venice, 45.45°N, 73.08°W, 19.IX.-11.X.1972, Dondale & Redner (1 ♂, CNC). **Ontario**: Moosonee, 51.24622°N, 80.67281°W, 17–20.VI.2010, NBP field party M1MP111 (1 ♀, LFC). USA: **New Hampshire: Coos Co.**, 8 mi S Gorham Pinkham Notch, 2000 feet, 11.IX.1987, J.M. Campbell & A. Davies, sifting Alnus litter and Sphagnum near pond (1 ♂, CNC).

##### Diagnosis.

This species may be distinguished by the following combination of characters: length 4.2–4.5 mm; body dark reddish-brown, with head dark brown, and legs and at least basal three antennal articles reddish-yellow; integument glossy; pronotum with dense punctation and pubescence; elytra with dense punctation and pubescence with very fine micro-granulation (Fig. 79); head subquadrate, slightly narrower than pronotum, large eyes, each about as long as postocular region in dorsal view (Fig. 79); pronotum subquadrate, widest at apical third (Fig. 79); elytra subparallel, as wide as pronotum and at suture about as long as pronotum (Fig. 79); abdomen subparallel, about as wide as elytra (Fig. 79); **Male.** Apical margin of tergite VIII with broad, moderate projection in middle three-fifth, with apical margin crenulate (Fig. 81); apical margin of sternite VIII broadly parabolic (Fig. 82); median lobe of aedeagus with tubus short and straight, apical part narrowly rounded in lateral view (Fig. 80). **Female.** Tergite VIII rounded apically (Fig. 83); apical margin of sternite VIII with broad, shallow median emargination (Fig. 84); spermatheca short, S-shaped, capsule short, club-shaped, stem broad, sinuate, slightly twisted posteriorly (Fig. 85).

This species may be distinguished from Liogluta castoris, Liogluta pseudocastoris, Liogluta microgranulosa, and Liogluta atriventris by the following combination of characters: antennae, pronotum and elytra reddish-yellow (Fig. 79); pronotum subquadrate (Fig. 79); shape of median lobe of aedeagus different in lateral view (Fig. 80); male tergite VIII with projection crenulate along apical margin (Fig. 81), spermatheca short, S-shaped with broad stem (Fig. 85).

##### Natural history.

Adults were collected in a conifer forest using pitfall traps, in a spruce-moss forest using carrion-baited traps, and in a highbush blueberry field. Others were collected by sifting litter and moss, sifting Alnus litter and Sphagnum moss near a pond, and treading flooded Carex and grasses. The flight period is from May to October.

##### Distribution.

Canada: **LB**, NF, **NS**, **QC**, **ON**. USA: **NH**.

#### Liogluta atriventris

Taxon classificationAnimaliaColeopteraStaphylinidae

(Casey, 1906)

Figs 86–89

Athetota atriventris Casey, 1906: 336. As Atheta (Liogluta): Bernhauer and Scheerpeltz 1926: 656; Moore and Legner 1975: 355. **Lectotype** (male): Canada, Victoria, Vancouver Island; atriventris Casey; Type USNM 39475; H.F. Wickham, Casey Bequest 1925 (USNM). Present designation.

##### Diagnosis

(based on male lectotype). This species may be distinguished by the following combination of characters: small body size, length 2.8 mm; head and abdomen dark brown, pronotum, elytra and legs reddish-yellow (Fig. 86); integument glossy with weak meshy microsculpture; pronotum and elytra with moderately dense punctation and pubescence, elytra with very fine micro-granulation (Fig. 86); head subquadrate, slightly narrower than pronotum; large eyes, each about as long as postocular region in dorsal view (Fig. 86); antennae of the holotype are partially damaged and cannot be completely described, but fifth and sixth articles suggest that missing funicle articles are subquadrate; pronotum slightly transverse, widest near the middle (Fig. 86); elytra wider and slightly longer than pronotum (Fig. 86); abdomen subparallel, about as wide as elytra (Fig. 86). **Male.** Apical margin of tergite VIII with broad, truncate projection in middle two-thirds bounded laterally by small tooth-like processes, apical margin crenulate (Fig. 88); apical margin of sternite VIII evenly broadly parabolic from base (Fig. 89); median lobe of aedeagus with tubus bent slightly ventrad at middle, apical part relatively broadly rounded in lateral view (Fig. 87). **Female.** Unknown.

*Liogluta atriventris* may be distinguished from the other species of the *granulosa* group by the following combination of characters: body size small, length 2.8 mm; elytra slightly longer than pronotum (Fig. 86); pronotum glossy with weak microsculpture (Fig. 86); shape of median lobe of aedeagus different in lateral view (Fig. 87), and projection on apical margin of male tergite VIII crenulate, with tooth-like processes laterally (Fig. 88).

##### Natural history.

Unknown.

##### Distribution.

Known only from Vancouver Island, British Columbia.

##### Comments.

This species is known only from one damaged male specimen. More specimens, including females, are needed for study to confirm the status of this species.

### *gigantea* species group

This group is characterized by: body broad, eyes large and bulging, diameter of eye about as long as postocular area of head in dorsal view (Fig. 90); integument of forebody glossy (Fig. 90); elytra not granulose, about one-fifth broader than pronotum, at suture about as long as pronotum (Fig. 90); apical margin of male tergite VIII rounded with broad crenulations (Fig. 92); median lobe of aedeagus with tubus arched slightly ventrad near apex, apical part narrow in lateral view (Fig. 91); spermatheca vaguely S-shaped (Fig. 96); female with apical margin of sternite VIII broadly truncate, with a row of microsetae (Fig. 95).

#### Liogluta gigantea

Taxon classificationAnimaliaColeopteraStaphylinidae

Klimaszewski & Langor, 2011

Figs 90–96

Liogluta gigantea Klimaszewski & Langor, in Klimaszewski et al. 2011: 167. **Holotype** (female): Canada, **Newfoundland**, Labrador, 75 km SW Goose Bay, Tr. 500, 53°02.6 N, 61°16.6 W, 13–26.VIII.2001, S. and J. Peck// Carrion trap, elevation 100 m, spruce-lichen forest, 2001-44 (LFC).

##### New locality data.

CANADA: **Québec**: 4 mi W Masham, near Mud Lake, 24.X.1967, J.M. Campbell & A. Smetana, Berlese sample ex lining of deserted beaver lodge (1 sex undetermined, CNC); Gatineau Park, near Mud Lake, 24.X.1967, A. Smetana (4 sex undetermined, CNC); **Ontario**: Rondeau Pr. Pk., Marsh Trail, 4.VI.1985, A. Davies & J.M. Campbell (1 ♂, CNC); Lake Superior Pr. Pk., Sand Riv., 6.VI.1973, J.M. Campbell & R. Parry (1 sex undetermined, CNC).

##### Diagnosis.

This species may be distinguished by: body length 4.2–5.0 mm, robust, broad, dark brown, with pronotum, elytra (except for scutellar region), and legs reddish-brown; forebody moderately glossy, with fine and dense punctation, short pubescence and meshed microsculpture (Fig. 90); head subquadrate, slightly narrower than pronotum, large eyes, each as long as postocular region in dorsal view (Fig. 90); antennae thin, all articles elongate to subquadrate (Fig. 90); pronotum transverse, widest at apical third (Fig. 90); elytra wider than pronotum, at suture as long as or slightly longer than pronotum, with posterior margin almost rectangular (Fig. 90); abdomen broad and flattened (Fig. 90). **Male (new description).** Apical margin of tergite VIII rounded with broad crenulations and small rounded process at middle (Fig. 92); apical margin of sternite VIII parabolic (Fig. 93); median lobe of aedeagus short and stout, with tubus arched slightly ventrad near apex, apical part narrow in lateral view (Fig. 91). **Female.** Apical margin of tergite VIII rounded-triangular (Fig. 94); apical margin of sternite VIII broadly truncate, with row of microsetae (Fig. 95); spermatheca vaguely S-shaped, capsule club-shaped, stem sinuate and twisted posteriorly (Fig. 96).

##### Distribution.

Canada: NF, **QC**, **ON**.

##### Natural history.

Adults were collected in June, August, and October, in carrion-baited pitfall traps in spruce forests, and from a Berlese funnel extraction of the interior of a deserted beaver lodge.

## Supplementary Material

XML Treatment for Liogluta

XML Treatment for Liogluta terminalis

XML Treatment for Liogluta trapezicollis

XML Treatment for Liogluta quadricollis

XML Treatment for Liogluta wickhami

XML Treatment for Liogluta vasta

XML Treatment for Liogluta nigropolita

XML Treatment for Liogluta nitens

XML Treatment for Liogluta granulosa

XML Treatment for Liogluta microgranulosa

XML Treatment for Liogluta castoris

XML Treatment for Liogluta pseudocastoris

XML Treatment for Liogluta intermedia

XML Treatment for Liogluta atriventris

XML Treatment for Liogluta gigantea
